# Configurable circular-polarization-dependent optoelectronic silent state for ultrahigh light ellipticity discrimination

**DOI:** 10.1038/s41377-023-01193-4

**Published:** 2023-07-14

**Authors:** Yonghao Bu, Xiansong Ren, Jing Zhou, Zhenhan Zhang, Jie Deng, Hangyu Xu, Runzhang Xie, Tianxin Li, Weida Hu, Xia Guo, Wei Lu, Xiaoshuang Chen

**Affiliations:** 1 State Key Laboratory of Infrared Physics, Shanghai Institute of Technical Physics, Chinese Academy of Sciences, 500 Yu Tian Road, 200083 Shanghai, China; 2 University of Chinese Academy of Sciences, 19 Yuquan Road, 100049 Beijing, China; 3 State Key Laboratory for Information Photonics and Optical Communications, School of Electronic Engineering, Beijing University of Posts and Telecommunications, 100876 Beijing, China

**Keywords:** Optoelectronic devices and components, Metamaterials, Nanophotonics and plasmonics

## Abstract

Filterless light-ellipticity-sensitive optoelectronic response generally has low discrimination, thus severely hindering the development of monolithic polarization detectors. Here, we achieve a breakthrough based on a configurable circular-polarization-dependent optoelectronic silent state created by the superposition of two photoresponses with enantiomerically opposite ellipticity dependences. The zero photocurrent and the significantly suppressed noise of the optoelectronic silent state singularly enhance the circular polarization extinction ratio (CPER) and the sensitivity to light ellipticity perturbation. The CPER of our device approaches infinity by the traditional definition. The newly established CPER taking noise into account is 3–4 orders of magnitude higher than those of ordinary integrated circular polarization detectors, and it remains high in an expanded wavelength range. The noise equivalent light ellipticity difference goes below 0.009° Hz^−1/2^ at modulation frequencies above 1000 Hz by a light power of 281 μW. This scheme brings a leap in developing monolithic ultracompact circular polarization detectors.

## Abstract (web summary)

Based on the configurable circular-polarization-dependent optoelectronic silent state, our integrated circular polarization detector achieves a near-infinite circular polarization extinction ratio and an ultralow noise equivalent light ellipticity difference of 0.009° Hz^−1/2^.

## Graphical abstract

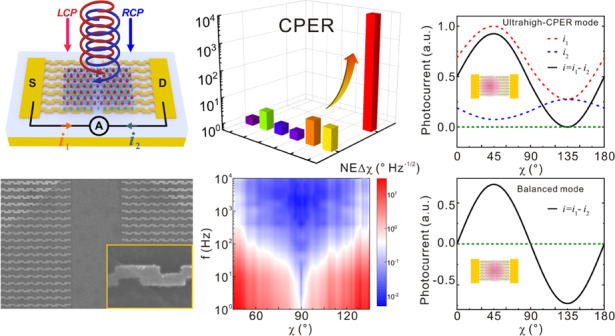

## Introduction

Polarization, as a primary physical quantity of light, is of great interest to almost all optical sciences and technologies^1–6^. Effective polarization detection with miniaturized devices is always sought after. Recently, monolithic polarization detectors have emerged as promising candidates^7–16^. Along with linear polarization detection, circular polarization (or light ellipticity) detection is essential for chiral molecule distinguishing^5,17^, vision dehazing^18^, magnetic field sensing^6^, quantum communication, and cryptography^19,20^. However, light ellipticity detection with conventional detectors is inherently difficult.

Traditional solutions rely on external optical systems containing polarizers and wave plates, unfavorably increasing the complexity and sizes of light ellipticity detectors. Although metasurfaces as flat optics could potentially shrink the footprints of light ellipticity detectors by replacing conventional polarizers or wave plates^8,14,21^, they work as additional filtering layers and thus inevitably cause energy loss and alignment difficulties. Materials with circular dichroism or circular photogalvanic effects have been proposed for filterless light ellipticity detection^16,22–24^. However, these materials are not common and their light ellipticity discrimination is quite low. In this situation, integrating plasmonic chiral structures directly with photodetection materials to realize compact light ellipticity detectors is progressively pursued^15,25–30^. The integrated plasmonic chiral structures not only provide circular polarization discrimination but also enhance the absorptances of the detection materials through an intensified local field^31–33^. However, the biggest issue is insufficient light ellipticity discrimination. The circular polarization extinction ratio (CPER), defined as the ratio of the responsivity for the light in the principle circular polarization state to that for the light in the orthogonal circular polarization state, is typically below 5. In comparison, the CPER of a conventional circular polarizer is usually above 1000. The key problem is that the photoresponse to the unwanted polarization cannot be efficiently suppressed.

In this work, the concept of optoelectronic silent state was adopted to address the insufficient light ellipticity discrimination of integrated circular polarization detectors. We propose and demonstrate an integrated circular polarization detector as a combination of two ordinary circular polarization detectors with enantiomerically opposite ellipticity dependences and opposite photoresponse polarities. The superposition of the two photoresponses leads to an optoelectronic silent state with zero photocurrent and significantly suppressed noise. The silent state can be set at various polarization states by tuning the magnitudes of the two photoresponses for superposition. A singularly high CPER or a singularly high sensitivity to light ellipticity change is realized when the silent state coincides with a circular polarization state or a chirality transition point (e.g., horizontal or vertical polarization). An ultracompact device of this kind is achieved. This work opens a new avenue to ultracompact solutions for high-performance polarization detection.

## Results

### Concept and principle

**Fig. 1:**
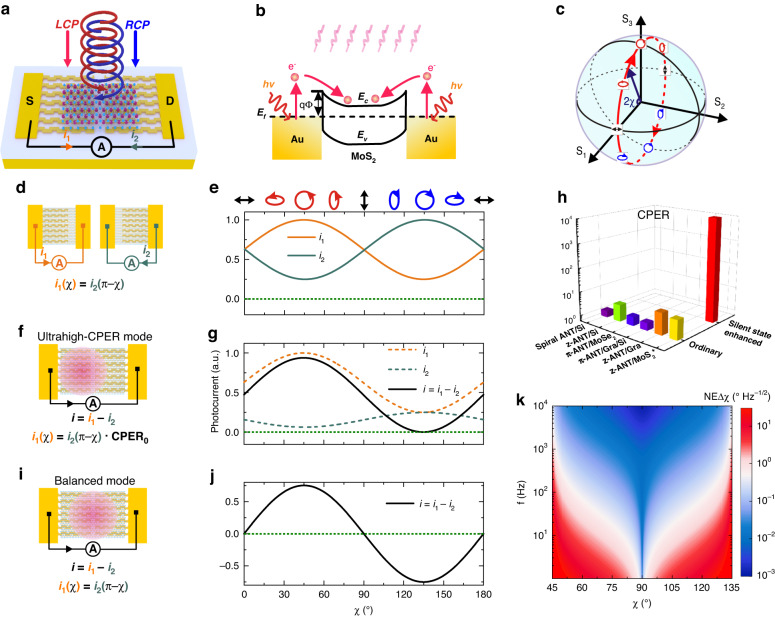
Structure and working principle of the silent-state-enhanced integrated circular polarization detector. **a** Device structure of an integrated circular polarization detector dimer based on the metal-MoS_2_-metal architecture. **b** Band diagram of the two metal-MoS_2_ junctions functioning as two Schottky diodes connected face-to-face; and illustration of hot electron injection. **c** Poincare sphere with the longitude line crossing the *S*_1_ and the *S*_3_ axes. As *χ* varies from 0 to *π*, the polarization state changes along a longitude line on the Poincare sphere and accomplishes a complete tour. **d** Diagrams of two ordinary integrated circular polarization detectors: one is integrated with the left-handed Z-antenna array on the left contact and the other with the right-handed Z-antenna array on the right contact. **e** Light ellipticity-dependent photocurrents of the two detector monomers. The photocurrents are based on the simulated absorptances. **f** Diagrams of the detector dimer in the ultrahigh-CPER mode. **g** Light ellipticity-dependent photocurrents from the two junction regions (*i*_1_, *i*_2_) and light ellipticity-dependent net photocurrent (*i* = *i*_1_ − *i*_2_) of the detector dimer in the ultrahigh-CPER mode. **h** Comparison of the CPERs of ordinary integrated circular polarization detectors with that of an integrated circular polarization detector dimer in the ultrahigh-CPER mode. The Z-antenna/MoS_2_ results are based on our detector dimer in the ordinary mode and that in the ultrahigh-CPER mode (*P*_light_ = 281 μW, *λ* = 1.48 μm, *f* = 333 Hz, Δ*f* = 1 Hz). The rest come from the literatures: spiral antenna/Si^25^, Z-antenna/Si^26^, π-antenna/MoSe_2_^27^, π-antenna/graphene/Si^15^, Z-antenna/graphene^28^. **i** Diagram of the detector dimer in the balanced mode. **j** Light ellipticity-dependent photocurrent of the detector dimer in the balanced mode. **k** Contour of NEΔ*χ* (*χ*, *f*) based on theoretical analysis

The silent-state-induced ultrahigh light ellipticity discrimination is demonstrated in an integrated circular polarization detector schematically shown in Fig. 1a. A piece of MoS_2_ bridges two metal contacts molded into plasmonic planar chiral structures. Plasmonic structures have been utilized to enhance the photoresponse of MoS_2_ detectors^34,35^. Recently, plasmonic planar chiral structures have been used to create circular polarization selectivity of light coupling and thus photoresponses^15,26–28^. In our device, the left contact is molded into a left-handed chiral structure to absorb left-handed circularly polarized (LCP) light and reflect right-handed circularly polarized (RCP) light. The right contact is molded into a right-handed chiral structure that behaves in an enantiomerically opposite manner. Two Schottky junction regions are formed at the two contacts, and they work as two Schottky photodiodes connected face-to-face (Fig. 1b). The flat-band Schottky barrier height is measured to be 161 meV through temperature dependent transfer characteristic test (Supplementary Note 1). At zero bias, the self-driven photocurrents are typically generated at the two junctions. In the near infrared range, the self-driven photocurrents at the MoS_2_-Au junctions are attributed to plasmonic resonance induced hot electron injection^36,37^. Light ellipticity is described by the ellipticity angle *χ*, which is half the angle between the polarization state vector and the equatorial plane of the Poincare sphere (Fig. 1c).

The silent-state-enhanced integrated circular polarization detector can be considered a detector dimer consisting of two detector monomers, which are two ordinary integrated circular polarization detectors with enantiomerically opposite ellipticity dependences and opposite photocurrent polarities (Fig. 1d). The left detector monomer is integrated with the left-handed chiral structure only at the left contact. Due to plasmonic enhancement, the self-driven photocurrent *i*_1_ is mainly generated from the left contact^32,33^, and thus controlled by the chiral structure. Thus, ${i}_{1}={P}_{l}\cdot {R}_{l}(\chi )$, where *P*_*l*_ denotes the light power received by the left photosensitive region and ${R}_{l}(\chi )$ the responsivity as a function of light ellipticity. When the plasmonic chiral structure takes the form of a Z-antenna array, the ellipticity-dependent *i*_1_ follows the curve in Fig. 1e. It reaches a maximum at *χ* = *π*/4 (LCP) and a minimum at *χ* = 3*π*/4 (RCP). The right detector monomer in Fig. 1d is just the enantiomer of the left one, so ${R}_{l}(\chi )={R}_{r}(\pi -\chi )$, where ${R}_{r}$ denotes the responsivity of the right photosensitive region in the right detector monomer. When the two detector monomers receive the same amount of power (*P*_*l*_ = *P*_*r*_), ${i}_{1}(\chi )={i}_{2}(\pi -\chi )$. The CPER of the left or right detector monomer is calculated to be ${i}_{1}(\pi /4)/{i}_{1}(3\pi /4)={i}_{2}(3\pi /4)/{i}_{2}(\pi /4)$. In this work, the CPER of either the two detector monomers is denoted as CPER_0_. For ordinary integrated circular polarization detectors, CPER equals CPER_0_.

When the two detector monomers are combined into a detector dimer (Fig. 1f), the two photosensitive regions of the monomers are inherited by the dimer. The self-driven photocurrent of the dimer can be considered as a difference between *i*_1_ and *i*_2_, i.e. $i={i}_{1}-{i}_{2}={P}_{l}\cdot {R}_{l}-{P}_{r}\cdot {R}_{r}$. Here, *i*_1_ (*i*_2_) denotes the self-driven photocurrent for the incident light only illuminating the left (right) photosensitive region, *P*_*l*_ (*P*_*r*_) the power received by the left (right) photosensitive region in the detector dimer, and *R*_*l*_ (*R*_*r*_) the corresponding responsivity. From another point of view, the two photoresponsive regions under illumination generate photoinduced electromotive forces (*ε*_1_ and *ε*_2_) with opposite polarities, and then the photocurrent *i* is driven by the total electromotive force (*ε*_1_ − *ε*_2_). According to circuit analysis, *ε*_1_ (*ε*_2_) is proportional to *i*_1_ (*i*_2_). The circuit model can be found in Supplementary Note 2. As the ratio of *i*_1_ to *i*_2_ varies from less than 1 to greater than 1, *i* experiences a polarity transition. When *i*_1_ = *i*_2_, the device reaches an optoelectronic silent state, corresponding to complete absence of collective charge motion. In this state, the device is equipotential since the opposite photoinduced electromotive forces at the two photosensitive regions are equal in magnitude. *i*_1_ and *i*_2_ can be adjusted by manipulating *P*_*l*_ and *P*_*r*_. Concerning a light spot illuminating both the two photosensitive regions (Fig. 1f), the power distribution over these two regions varies with the light spot position (Supplementary Note 3). At the position where ${P}_{l}={P}_{r}\cdot {{\rm{CPER}}}_{0}$, the incident light induces zero photocurrent at *χ* = 3*π*/4, indicating that the device is in the silent state under RCP illumination (Fig. 1g). Consequently, the traditional CPER of the detector dimer approaches infinity. In fact, the “zero photocurrent” at RCP ($i(3\pi /4)$) cannot be absolutely zero, and the down limit is the noise (*i*_noise_). Then, the CPER is redefined as the ratio of the photoresponse under LCP illumination to the noise under RCP illumination:

1 $${\rm{CPER}}=\frac{{i}_{1}(\pi /4)}{{i}_{noise}}(1-1/{{\rm{CPER}}}_{0}^{2})$$

*i*_noise_ is defined as $\sqrt{{\int }_{{f}_{1}}^{{f}_{2}}{S}_{i-noise}df}$, where *S*_*i*-noise_ is the noise spectral density, and *f* denotes the modulation frequency. A detailed derivation of Eq. 1 can be found in Supplementary Note 4. This CPER is proportional to the signal-to-noise ratio (SNR) and positively correlated with CPER_0_. If the noise under RCP illumination tends to zero, the CPER indeed approaches infinity. Then, the detector dimer can distinguish LCP and RCP light with the highest contrast no matter how weak the signal is. This technical leap is surely going to revolutionize polarization detection and its broadband applications. It is interesting to note that the noise of the dimer does experience a cancellation in the silent state due to the complete absence of photocurrent and dark current. The noise includes the 1/f noise, the light intensity noise, the generation-recombination (G-R) noise, the shot noise, and the thermal noise. Concerning 1/f noise, although there is no consensus on the source of this type of noise, it manifests itself as fluctuations in electrical conductance in electronic devices^38^. Thus, this noise decreases with the device current, and finally vanishes at the silent state corresponding to zero current. The light intensity noise currents generated from the left and the right photosensitive regions are equal in magnitude and opposite in polarity, leading to cancellation in the silent state. Although the last three types of noise are not reduced in the silent state, they are usually much smaller. Therefore, the elimination of the 1/f noise and the light intensity noise in the silent state leads to ultralow noise. A detailed noise analysis can be found in Supplementary Note 5. As a result, the CPER of our device is as high as 1.07 × 10^4^, more than three orders of magnitude higher than those of ordinary integrated circular polarization detectors (Fig. 1h). Since the silent state gives rise to cancellation of photocurrent and noise, when it appears under LCP or RCP illumination, the light ellipticity discrimination of the detector dimer becomes singularly high, and this operation mode is called an ultrahigh-CPER mode. In addition to our scheme, vectorial photoresponse manipulation in a semimetallic detection material could be another way to obtain an ultrahigh polarization extinction ratio in an integrated polarization detector^10,12^.

The detector dimer can also sensitively measure small changes in light ellipticity in the balanced mode (Fig. 1i, j). In this mode, *P*_*l*_ = *P*_*r*_, so ${i}_{1}(\chi )={i}_{2}(\pi -\chi )$ and then the silent state appears at chirality transition points of the incident light, i.e., horizontal polarization (HP) and vertical polarization (VP), as illustrated in Fig. 1j. At a chirality transition point, due to the maximum derivative of the photoresponse with respect to *χ* and the near-zero noise caused by the silent state, the sensitivity to light ellipticity change ($(di/d\chi )/{i}_{noise}$) divergently increases to a singularity. The noise equivalent light ellipticity difference (${\rm{NE}}\varDelta \chi ={i}_{noise}/(di/d\chi )$) is adopted to represent the smallest appreciable change in light ellipticity. As shown in Fig. 1k, NEΔ*χ* at the chirality transition point (*χ* = *π*/2) is three to four orders of magnitude smaller than that near *χ* = *π*/4 or 3*π*/4 in the low-frequency range. NEΔ*χ* is inversely proportional to SNR. Since the 1/f noise and the G-R noise decrease with the increasing *f*, NEΔ*χ* improves with the increasing *f*. Moreover, the portion of the 1/f noise in the total noise decreases with the increasing *f*, so the noise at the *χ* angles other than the chirality transition point decreases faster, and then the *χ* range of ultrasmall NEΔ*χ* expands. This range is defined as $2|{\chi }_{r}-90^\circ |$, where ${\chi }_{r}$ is determined by ${\rm{NE}}\varDelta \chi ({\chi }_{r})=2\cdot {\rm{NE}}\varDelta \chi (90^\circ )$. In addition, the device in the balanced mode can distinguish the chirality of the incident light regardless of whether it is circularly polarized or elliptically polarized. As shown in Fig. 1j, the photocurrent is positive for any left-handed polarization and negative for any right-handed polarization.

The detector dimer can operate in the ultrahigh-CPER mode for ultrahigh discrimination of ellipticity, in the balanced mode for ultrahigh sensitivity to ellipticitiy perturbation, and in the ordinary mode, where the light spot is thoroughly shifted to the left or right photosensitive region and the dimer behaves like the monomer shown in Fig. 1d.

### Photoresponse of Z-antenna integrated MoS_2_

**Fig. 2:**
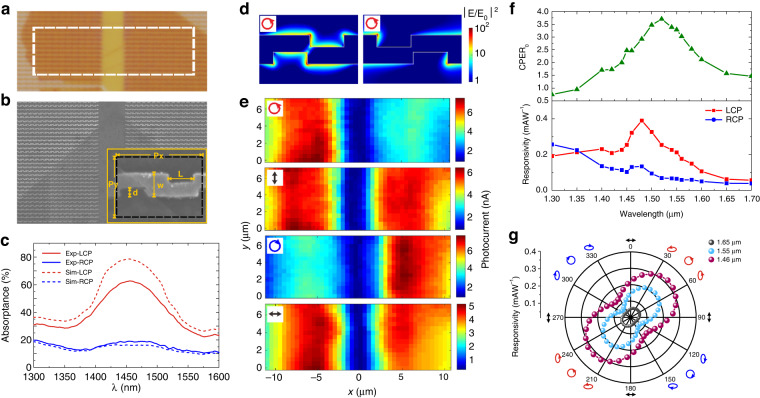
Optical and optoelectronic properties of the Z-antenna-integrated MoS_2_. **a** Microscopic photo picture of the integrated circular polarization detector dimer. The white dashed frame corresponds to the area for laser induced photocurrent mapping. The thickness of the MoS_2_ measured by AFM is about 8 nm (Supplementary Note 11). **b** SEM image of the device. Inset: zoomed-in image of a single Z-antenna at the edge of the MoS_2_ film. The dimensions of the Z-antenna: *P*_*x*_ = 780 nm and *P*_*y*_ = 550 nm represent the horizontal and vertical periods, *w* = 220 nm, *d* = 90 nm and *L* = 280 nm. **c** Measured and simulated absorptance spectra of the Z-antenna array under LCP and RCP illumination. **d** Light field (|*E*/*E*_0_ | ^2^) distributions in the MoS_2_ plane on top of the Z-antenna array under LCP and RCP illumination. The wavelength is 1.55 μm. *E*_0_ denotes the amplitude of the incident light. **e** Laser induced photocurrent mapping results under LCP, VP, RCP, and HP illumination, respectively. *P*_light_ = 36 μW. *f* = 333 Hz. **f** Spectra of CPER_0_, and spectra of responsivity induced by LCP and RCP light, respectively. **g** Ellipticity angle dependent responsivity polar diagrams at the wavelengths of 1.46 μm, 1.55 μm, and 1.7 μm, respectively

A microscopic picture of our device is shown in Fig. 2a. The plasmonic planar chiral structure is a Z-antenna array, as shown in the SEM image (Fig. 2b). The Z-antenna together with the Al_2_O_3_ (200 nm)/Au cavity couples the incident light into a surface plasmon polariton (SPP) wave in an ellipticity-discriminative manner^26,39^. The light ellipticity discrimination is attributed to the twisted metal strips and the cavity. The former scatters light into polarization unconverted field and polarization converted field. The interference of them induces different SPP excitation efficiencies for LCP and RCP light. The latter enlarges the difference through multiple reflection interference. Concerning the left-handed Z-antenna, LCP light efficiently excites the SPP and enhances light absorption, while RCP light is largely reflected (Fig. 2c). The ellipticity dependence of the right-handed Z-antenna is the opposite. Thus, under LCP or RCP illumination, there is a contrast between the left- and right-handed Z-antenna arrays in terms of local field intensity (Fig. 2d) and photocurrent (Fig. 2e). Under HP or VP illumination, the photocurrents from the two Z-antenna arrays are approximately the same. The scheme of using cavity coupled Z-antenna array to create circular polarization discrimination can be applied to many detection materials such as traditional semiconductors^26^, 2D materials^28^, and van der Waals heterostructures^40,41^. In the near infrared range, the photocurrent generation at the MoS_2_-metal junction is attributed to hot electron injection^36,37^. Nevertheless, the silent-state-induced ultrahigh discrimination of light ellipticity is not restricted to hot electron injection. It can be applied to various detection materials, device structures, and photoresponse mechanisms (Supplementary Note 6).

The responsivity spectra measured at the left-handed Z-antenna array (Fig. 2f bottom row) are consistent with the absorptance spectra (Fig. 2c). The plasmonic resonance enhances the absorptance and thus the responsivity. The spectrum of CPER_0_ forms a peak around the SPP resonance. The peak value is 3.77. The responsivity was also measured as a function of *χ* (Fig. 2g). At the three wavelengths for measurement, the responsivity follows the same ellipticity dependence. It reaches a maximum at *χ* = 45° or 225° (LCP) and a minimum at *χ* = 135° or 315° (RCP). The routine electrical and optoelectronic characteristics of our device can be found in Supplementary Note 7.

### Ultrahigh-CPER mode

**Fig. 3:**
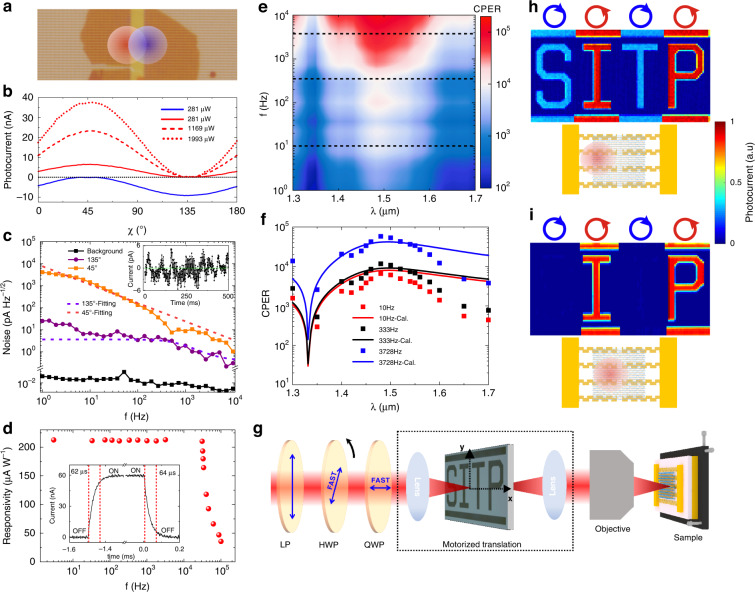
Characterization of the device in the ultrahigh-CPER mode. **a** Microscopic photo picture of the integrated circular polarization detector dimer in the ultrahigh-CPER mode. The red and blue circles indicate the light spot positions for the detector dimer in the LCP-responsive and RCP-responsive ultrahigh-CPER mode, respectively. **b** Ellipticity angle dependent photocurrents of the detector dimer in the LCP-responsive ultrahigh-CPER mode and in the RCP-responsive ultrahigh-CPER mode at different light power. **c** Noise current spectra of the detector dimer in the LCP-responsive ultrahigh-CPER mode at *χ* = 45° (LCP) and *χ* = 135° (RCP, silent state). Both measured data and fitted results based on a theoretical noise model are presented. The black squares denote the background noise of the instrument. Inset: photocurrent at *χ* = 135° in real time. **d** Responsivity of the detector dimer as a function of modulation frequency. Inset: waveform at the modulation frequency of 333 Hz. The rise time and the fall time, defined as the time for the photocurrent to change between 10% and 90% of the maximum value, are 62 μs and 64 μs, respectively. **e** Contour of CPER (*λ*, *f*) of the detector dimer in the LCP-responsive ultrahigh-CPER mode based on experimental data. *P*_light_ = 281 μW. **f** CPER spectra (squares) at three modulation frequencies extracted from **e** at the dashed lines, accompanied by the calculated results (solid lines) based on the simulated photoresponses and fitted noise spectra. **g** Diagram of the light ellipticity-dependent single-pixel imaging setup. **h** Light ellipticity-dependent imaging result in the ordinary circular polarization detection mode, where only the left photosensitive region of the detector dimer is illuminated by the incident light. *P*_light_ = 500 μW. *f* = 333 Hz. The blue or the red circle on top of the image marks out RCP or LCP light that sweeps over the corresponding domain. **i** Light ellipticity-dependent imaging result of the detector dimer in the LCP-responsive ultrahigh-CPER mode. The diagrams in (**h**) and (**i**) indicate the positions of the light spot

As shown in Fig. 3a, b, when the light spot is located at the red circle, the detector dimer works in the LCP-responsive ultrahigh-CPER mode, where *i* becomes zero at *χ* = 135° (RCP) and reaches a maximum at *χ* = 45° (LCP) (Fig. 3b red curves). As light power increases, *i* remains zero at *χ* = 135° and increases proportionally at other *χ* angles. When the light spot shifts to the blue circle, the detector dimer works in the RCP-responsive ultrahigh-CPER mode, where *i* becomes zero at LCP and reaches a maximum absolute value at RCP (Fig. 3b blue curve). Without loss of generality, we discuss only the ultrahigh-CPER mode for LCP in the following. As shown in Fig. 3c, the “zero photocurrent” at *χ* = 135° fluctuates due to noise. Since the device is in the silent state at *χ* = 135°, the noise at this *χ* angle is on average two orders of magnitude lower than that at *χ* = 45° in the 1–1000 Hz frequency range due to the elimination of the 1/f noise and the light intensity noise. The significant reduction in noise at *χ* = 135° evidences that the silent state corresponds to a true zero-photocurrent situation. In comparison, if the two detector monomers are not combined into a dimer but separately connected to a computer performing the calculation of *i*_1_−*i*_2_, there is no reduction in noise in the *i* = 0 state that is not pure silent (Supplementary Note 5). The light intensity fluctuation in our experiment is smaller than 0.01%, so it is minor compared to the 1/f noise. Nevertheless, it can be much more severe in some cases, such as gas or fluid detection^42^. Since the noise decreases with the increasing *f*, both the SNR and the CPER improve with the increasing *f* as long as the device can keep up with the fast modulation. The 3 dB modulation bandwidth of our device is ~30 kHz (Fig. 3d), indicating that the CPER continuously improves within this band.

As shown by the contour of CPER (*λ*, *f*) (Fig. 3e), the highest CPER within the *λ* (1.36–1.7 μm)-*f* (1–10k Hz) region is ~1.5 × 10^5^, and the lowest is still higher than 120. The wavelength dependence of the CPER follows the similar trend of the responsivity spectrum and the CPER_0_ spectrum, which is consistent with Eq. 1. The power distribution is adjusted according to the CPER_0_ at each wavelength to ensure *i* = 0 under RCP illumination. Although the CPER_0_ at an off-resonance wavelength such as 1.7 μm is as small as 1.47, the CPER still exceeds 120. Therefore, the CPER remains at a high level over a wavelength range much wider than the resonance of the Z-antenna. As expected, the CPER increases with the increasing *f* throughout this modulation band, since the device is fast enough for these modulation frequencies. Figure 3f confirms that the experimental CPER agrees with the theoretical result. To demonstrate the advantage of the enhanced CPER, we conducted a light ellipticity-dependent single-pixel imaging experiment. The imaging setup is diagrammed in Fig. 3g. A mask with a transparent “SITP” pattern was inserted in the optical path. As the laser sweeps over the mask, the detector receives spatially variant power and generates an image. When the laser scanned over the “S” domain, the light was RCP. Then, the polarization state switched between LCP and RCP alternatively for the “I”, “T”, and “P” domains. When the detector dimer operates in the ordinary mode, the RCP signal (“S” and “T”) cannot be adequately suppressed (Fig. 3h) since the CPER of the ordinary mode is only 3.3. In comparison, in the ultrahigh-CPER mode, the “S” and “T” completely disappear (Fig. 3i).

### Balanced mode

**Fig. 4:**
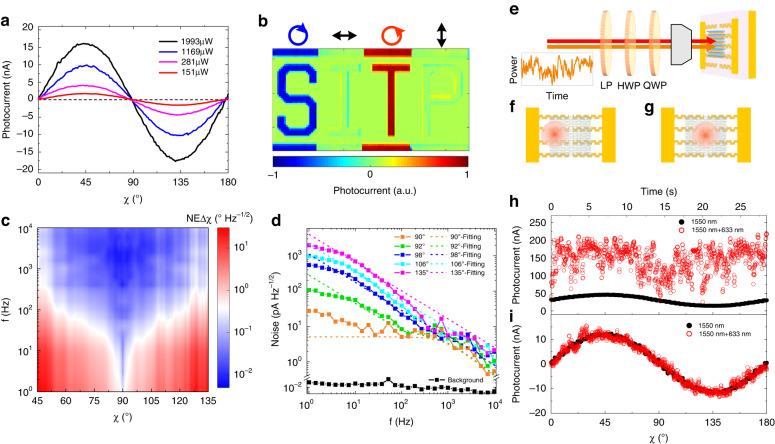
Characterization of the device in the balanced mode. **a** Ellipticity angle dependent photocurrents at different light power in the balanced mode. **b** Light ellipticity-dependent imaging in the balanced mode. **c** Contour of NEΔ*χ* (*χ*, *f*) in the balanced mode. *P*_light_ = 281 μW. **d** Noise current spectra at various ellipticity angles from 90° to 135° in the balanced mode, accompanied by fitted spectra based on a theoretical noise model. The black squares denote the background noise of the instrument. **e** Diagram of the anti-disturbance test of our device in the balanced mode. The red arrow denotes the infrared light with a wavelength of 1.55 μm. The orange arrow denotes the visible light with a wavelength of 0.63 μm. The two laser beams shares the same optical path. The visible light is randomly fluctuated mimicking a disturbance. **f**, **g** Diagrams of our device illuminated by the infrared light (signal) and the visible light (disturbance) in the ordinary mode and the balanced mode, respectively. **h**, **i** Photocurrent varying with time and ellipticity angle in the ordinary mode, and that in the balanced mode. The ellipticity angle controlled by a rotating HWP is correlated with time. The black solid circles in (**h**, **i**) represent the photocurrent induced by the signal light only. The red hollow circles in (**h**, **i**) represent the photocurrent induced by both the signal and the disturbance light

In the balanced mode, the silent state appears at chirality transition points such as *χ* = 0° (HP) and *χ* = 90° (VP) (Fig. 4a). The polarity of the photocurrent indicates the polarization chirality of the incident light: *i* > 0 (*i* < 0) for left-handed (right-handed) polarizations (Fig. 4a, b). The device is very sensitive to light ellipticity perturbation around the chirality transition points due to the maximum $di/d\chi$ and the singularly low noise caused by the silent state. As shown by the contour of NEΔ*χ* (*χ*, *f*) (Fig. 4c), NEΔ*χ* exhibits a sharp improvement around the chirality transition point *χ* = 90° in the low-frequency range. This is consistent with the rapid decrease in the noise as *χ* approaches 90°, where the device reaches the silent state (Fig. 4d). When *χ* deviates from the chirality transition point by only 2°, the noise increases by 3.33 times on average within the 1–100 Hz frequency range. In this range, NEΔ*χ* at *χ* = 90° is hundreds of times smaller than that near *χ* = 45° or 135°. As *f* increases, the noise decreases, and the proportion of the 1/f noise also decreases, so NEΔ*χ* improves at all *χ* angles, and the *χ* range of ultrasmall NEΔ*χ* expands. From 1 Hz to 10,000 Hz, the NEΔ*χ* averaged over the *χ* angles improves from 7° Hz^−1/2^ to 0.06° Hz^−1/2^. The NEΔ*χ* at *χ* = 90° goes below 0.009° Hz^−1/2^ at frequencies above 1000 Hz. Moreover, the *χ* range of ultrasmall NEΔ*χ* increases from 1.7° to 32°. Since NEΔ*χ* is inversely proportional to SNR, increasing the light power or enhancing the responsivity also benefits NEΔ*χ*.

Moreover, the balanced mode can prevent common mode disturbances. As shown in Fig. 4e, a near infrared signal beam (*λ* = 1.55 μm) and a visible jamming beam (*λ* = 0.63 μm) were simultaneously focused on the device at the same position. The photocurrent fluctuation caused by the disturbance is 5 times larger than the signal. During the test, the photocurrent was recorded in real time, and the ellipticity angle controlled by a rotating HWP was correlated with time. In the ordinary mode, the signal was drowned in the disturbance (Fig. 4h). In the balanced mode, the visible disturbance induces zero photocurrent at all *χ* angles since the Z-antenna is insensitive to the ellipticity of the visible light. As a result, the signal showed high fidelity (Fig. 4i). When the polarization state of the jamming light is fixed at a chirality transition point, even though the wavelength is within the ellipticity sensitive range of the Z-antenna, the intensity fluctuation can still be eliminated (Supplementary Note 8).

## Discussion

Compared to ordinary integrated circular polarization detectors, the silent-state-enhanced integrated circular polarization detector has several distinguished features: ultrahigh CPER in an expanded wavelength range, ultrahigh sensitivity to light ellipticity perturbation, and immunity against common mode disturbances. With these features, the silent-state-enhanced integrated circular polarization detector is comparable to or even surpasses traditional circular polarization detectors (Supplementary Note 9), without compromising ultracompact size and high speed.

Moreover, the silent-state scheme is not restricted to a specific detection material, wavelength range, photoresponse mechanism, or detector structure. As long as two light ellipticity-dependent self-driven photocurrent sources with opposite handednesses are connected face-to-face, the silent-state scheme can be implemented (Supplementary Note 6). A graphene version of the silent-state-enhanced integrated circular polarization detector was fabricated and characterized. Both the ultrahigh-CPER mode and the balanced mode are achieved in this device, and the maximum responsivity is almost 20 times higher than that of the MoS_2_ device (Supplementary Note 10). Therefore, the applicability of this scheme is expected to be very broad.

In addition to the power distribution, the magnitudes of the photoresponses from the left and the right photosensitive regions can also be adjusted by using a split gate to control the responsivities. The tuning range can be larger than 40 dB (Supplementary Note 7).

The CPER and the NEΔ*χ* can both be improved by shortening the channel length, since the light spot illuminates more photosensitive areas and the SNR rises. In our device, when the channel length decreases from 4 μm to 1 μm, the power received by the photosensitive regions is enhanced by 41% in the ultrahigh-CPER mode and enhanced by 50% in the balanced mode (Supplementary Note 3). Therefore, the CPER and the NEΔ*χ* improve 41% and 50%, respectively.

In summary, we achieved ultrahigh light ellipticity discrimination in a monolithic ultracompact circular polarization detector based on a configurable circular-polarization-dependent optoelectronic silent state with zero photocurrent and significantly suppressed noise. The silent state is created by the superposition of two photoresponses with enantiomerically opposite ellipticity dependences. This scheme is generally applicable to various detection materials and device structures. The silent-state-enhanced integrated circular polarization detector has been demonstrated as a promising upgrade of ordinary integrated circular polarization detectors and already shows great potential to replace traditional circular polarization detectors.

## Materials and methods

### Device fabrication

The bottom film (Ti/Au/Ti, 10/100/5 nm) was deposited on a SiO_2_ (285 nm)/Si substrate by electron beam evaporation. Then, a 200 nm thick Al_2_O_3_ layer was deposited on the metal plane by plasma-enhanced atomic layer deposition at 250 °C. The two contacts with Z-antenna array were created by a lift-off process. The pattern was defined by electron beam lithography. Then, a Ti/Au (3/27 nm) thin film was deposited by electron beam evaporation. After that, by striping the resist, the contacts with Z-antenna array were achieved. MoS_2_ pieces were mechanically peeled off the bulk MoS_2_ single crystal with a Scotch tape, and then transferred to a SiO_2_/Si substrate for selection. After inspection under a microscope, a proper piece of MoS_2_ was picked up and transferred onto the semi-finished device by polyvinyl alcohol (PVA) and polydimethylsiloxane (PDMS) through a high-precision two-dimensional material transfer platform. The MoS_2_ piece bridges the two Z-antenna arrays across the 4 μm channel. Finally, the residual PVA film was removed by deionized water. The thickness of the MoS_2_ piece was characterized by AFM.

### Optoelectronic characterization

The light ellipticity-dependent photocurrent test and single-pixel imaging were conducted through a home-built polarization controlled microscopic optoelectronic characterization system, as diagrammed in Fig. 3g. The device to be tested was placed on an X-Y stepper motor stage (LNR502E, Thorlabs). The laser beam generated by a laser diode (1550 nm and 633 nm, Thorlabs) was focused on the device by a 50X (or 5X) NIR objective (Mitutoyo). A supercontinuum laser (YSL) with an acousto-optic tunable filter was employed to measure the photoresponse spectrum. The laser power was modulated by a function generator. The polarization state of the incident light was controlled by a linear polarizer (LP), a half-wave plate (HWP), and a quarter-wave plate (QWP) inserted in the parallel light path before the objective. The LP is aligned in the *y*-direction. The fast axis of the QWP is aligned in the *x*-direction. As the HWP between the LP and the QWP rotates, the polarization state varies along the longitude line crossing the *S*_1_ and the *S*_3_ axis on the Poincare sphere. The angle between the linear polarization and the fast axis of the HWP equals *χ*/2. The HWP was automatically rotated by a motorized rotation mount. The parts within the dashed frame in Fig. 3g are for the imaging experiment, and they were removed during the photocurrent tests. For the photocurrent mapping, the incident light was focused into a spot about 2.5 μm in diameter.

The photocurrent was collected by a digital source meter (B2912A, Keysight) or a lock-in amplifier (SR830, Stanford) combined with a current preamplifier (SR570, Stanford). The motorized stages and the measuring instruments were collaboratively controlled by a computer to achieve photocurrent mapping and single-pixel imaging. The 3 dB modulation bandwidth of our device was extracted from the modulation frequency dependent responsivity obtained through the preamplifier and the lock-in amplifier. The rise time and the fall time of the photoresponse were characterized by an oscilloscope (MSO54, Tektronix). The noise test was carried out by the lock-in amplifier (SR860, Stanford). The equivalent noise bandwidth (ENBW) of the lock-in amplifier was set to be 1/(4*T*). The filter slope was set to be 6 dB oct^−1^. The time constant (*T*) was set to be 100 ms. The I–V test and the transferred characteristic test were conducted by a parameter analyzer (4200A, Keithley).

### Absorptance measurement

The absorptance was considered as one minus reflectance since the transmitting light was blocked by the metal plane at the bottom. The reflectance measurement wave conducted through a microscope and a spectrometer. The light source was a halogen lamp. The light was concentrated on the device through the objective. The polarization state was controlled by a LP and a QWP. By adjusting the QWP, the incident light was set as LCP or RCP. An optical fiber spectrometer is used to collect the reflected light.

### Theoretical analysis

During the calculation of the photocurrent $i={P}_{l}\cdot {R}_{l}(\chi )-{P}_{r}\cdot {R}_{r}(\chi )$, where *R*_*l*_ (*χ*) and *R*_*r*_ (*χ*) are based on the light absorptances of the left and the right photosensitive regions, respectively, and normalized by our experimental data: *R*_*l*_ (*π*/4) = *R*_*r*_ (3*π*/4) = 0.2 mA W^−1^. *P*_*l*_ and *P*_*r*_ are calculated as the power received by the left and the right junction regions, respectively (Supplementary Note 2). *P*_light_ = 281 μW. The noise spectra are based on theoretical expressions fitted to our experimental results. The noise current is based on the theoretical expressions fitted to the experiment results. Lumerical FDTD-Solutions and Comsol Multiphysics were employed to simulate the optical properties of the Z-antenna integrated MoS_2_.

## Supplementary information

Supplementary Information
