# Supplementary material for: Configurable circular-polarization-dependent optoelectronic silent state for ultrahigh light ellipticity discrimination
Source: Light Sci Appl. 2023 Jul 14;12:176. doi: 10.1038/s41377-023-01193-4 (PMC10345193; doi:10.1038/s41377-023-01193-4)
Supplement: Supplementary file 1 — Supplementary Information [file 41377_2023_1193_MOESM1_ESM.docx]

# Supplementary Information for

# Configurable circular-polarization-dependent optoelectronic silent state for ultrahigh light ellipticity discrimination

Yonghao Bu^1, 2, ‡^, Xiansong Ren^1, 3, ‡^, Jing Zhou^* 1, 2, ‡^, Zhenhan Zhang^1, ‡^, Jie Deng^1, 2^, Hangyu Xu^1, 2^, Runzhang Xie^1^, Tianxin Li^1, 2^, Weida Hu^* 1, 2^, Xia Guo^3^, Wei Lu^1, 2^, Xiaoshuang Chen^* 1, 2^

^1^ State Key Laboratory of Infrared Physics, Shanghai Institute of Technical Physics, Chinese Academy of Sciences, 500 Yu Tian Road, Shanghai, 200083, China^2^ University of Chinese Academy of Sciences, 19 Yuquan Road, Beijing, 100049, China

^3^ State Key Laboratory for Information Photonics and Optical Communications, School of Electronic Engineering, Beijing University of Posts and Telecommunications, Beijing 100876, China

‡ These authors contributed equally: Yonghao Bu, Xiansong Ren, Jing Zhou, Zhenhan Zhang.

^*^ Corresponding authors: Jing Zhou, Weida Hu, Xiaoshuang Chen.

**This file includes:**

Supplementary Notes 1 to 11

Figs. S1 to S15

Table S1

## Note 1. The measurement of Schottky barrier height

We quantitatively measured the Schottky barrier height through temperature dependent transfer characteristic test. Basically, our device is considered as a pair of back-to-back Schottky diodes, where most of the voltage drop occurs on the reverse bias side (Fig. S1a). Thus, the electrical transport across a Schottky contact into MoS_2_ can be described by the 2D thermionic emission equation^1-5^:

(S1)

where *I*_ds_ is the device current, *A* is the junction area, $A_{2d}^{*}$is the 2D equivalent Richardson constant, *k* is the Boltzmann constant, and *T* is the temperature. Under the reverse bias condition (*V*_ds_ = −1 V), $-qV_{ds}\ll kT$, so $\exp\left(qV_{ds}/kT\right)-1\simeq-1$. In this case, the Schottky barrier *Φ*_SB_ can be extracted from the Arrhenius plots diagram based on the following equation:

$\ln\left(-\frac{I_{ds}}{T^{3/2}}\right)=-\frac{q\Phi_{SB}}{kT}+c$ (S2)

where *c* is a constant and *Φ*_SB_ is the slope in the Arrhenius diagram. Fig. S1b shows the temperature dependent transfer characteristic curves. Fig. S1c shows the Arrhenius plots with linear fittings. Based on Eq. S2, the Schottky barrier height as a function of gate voltage (*V*_g_) is extracted as shown in Fig. S1d. The Schottky barrier height was determined from the flat-band voltage, which is defined as the gate voltage at which the curve ceases to decrease linearly with the gate voltage^1-5^. In this way, the flat-band Schottky barrier height of our device is extracted to be 161 meV, which is in agreement with previous studies^1-5^. During the experiment, the device was placed in a variable temperature Dewar, and the measurement was conducted in the dark. The transfer characteristic curves at different temperatures were measured by a digital source meter (B2912A, Keysight). The extracted Schottky barrier height is smaller than the difference between the work function of Au and that of MoS_2_ probably due to the pinning effect^4^.


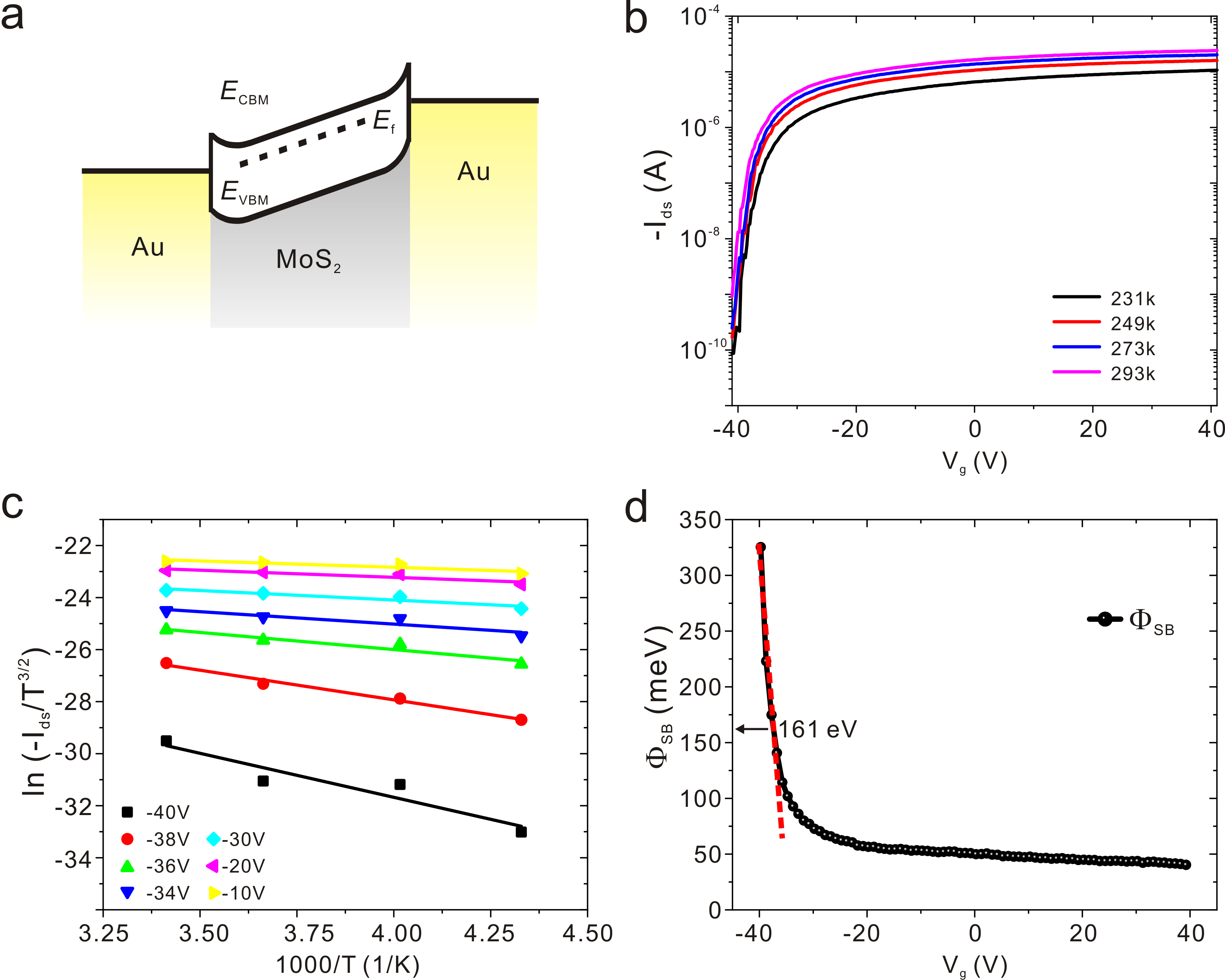


**Fig. S1** **a,** Schematic diagram of the integrated circular polarization detector based on the metal-MoS_2_-metal architecture and the MoS_2_ energy band distribution under a reversed bias. **b,** Transfer characteristic curves at different temperatures. **c**, The Arrhenius plots at different gate voltages with linear fittings. **d**, Schottky barrier height as a function of the gate voltage. The flat-band Schottky barrier height is extracted to be 161 meV.

## Note 2. Circuit model for the silent state enhanced integrated circular polarization detector

The two photoresponsive regions under illumination generate photoinduced electromotive forces (*ε*_1_ and *ε*_2_) with opposite polarities. They can be modeled as two photoinduced voltage sources with internal resistances *r*_1_ and *r*_2_, respectively. Thus, the device can be modeled as an effective circuit, shown in Fig. S2.

The photoinduced electromotive force *ε*_1,2_ is proportional to the light power and follows the light ellipticity dependence controlled by the chiral structure:

$\varepsilon_{1}=P_{l}\cdot R_{V-l}\left(\chi\right)$ (S3)

$\varepsilon_{2}=P_{r}\cdot R_{V-r}\left(\chi\right)$ (S4)

where *P_l,r_* denotes the light power received by the left or right photosensitive region, and $R_{V-l,r}\left(\chi\right)$ is the light ellipticity-dependent photovoltage responsivity of the left or right photosensitive region. Then, the photocurrent for the incident light only illuminating the left or right photosensitive region writes

$i_{1}=\varepsilon_{1}/\left(r_{1}+r_{ch}+r_{2}\right)$ (S5)

$i_{2}=\varepsilon_{2}/\left(r_{1}+r_{ch}+r_{2}\right)$ (S6)

where *r*_ch_ denotes the resistance of the channel. *i*_1_ and *i*_2_ can also be expressed as the product of the light power and the photocurrent responsivity ($R_{I-l,r}\left(\chi\right)$), i.e.

$i_{1}=P_{l}\cdot R_{I-l}\left(\chi\right)$ (S7)

$i_{2}=P_{r}\cdot R_{I-r}\left(\chi\right)$ (S8)

Based on this model, the superposition of the two photoresponses can be regarded as the superposition of two photoinduced voltage sources in series, and also as the superposition of *i*_1_ and *i*_2_. The total photovoltage of the device equals *ε*_1_ − *ε*_2_, and the total self-driven photocurrent *i* equals *i*_1_ − *i*_2_.


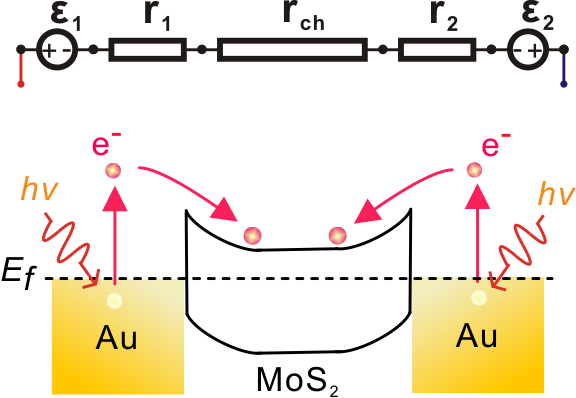


**Fig. S2.** Diagram of the circuit model for the silent state enhanced integrated circular polarization detector.

## Note 3. Power distribution by shifting the light spot

**3.1 Light spot profile modeling**

The light spot was measured by a Scanning-Slit Optical Beam Profiler (Thorlabs BP209-IR/M), and the profile along the *x* axis is shown by the black scatters in Fig. S3a. The light spot can be described by a two-dimensional Gaussian function:

$P_{0}\cdot\exp\left(\frac{-x^{2}-y^{2}}{c^{2}}\right)$ (S9)

where *P*_0_ represents the optical power density at the center of the spot. The constant *c* is determined to be 5.309 μm based on the measured profile. As shown in Fig. S3a, the Gaussian function (red line) agrees well with the measured profile. The total power of the light spot equals:

$P_{light}=\int_{-\infty}^{+\infty} \int_{-\infty}^{+\infty} P_{0}\exp\left(\frac{-x^{2}-y^{2}}{c^{2}}\right)dxdy=P_{0}c^{2}\pi$ (S10)


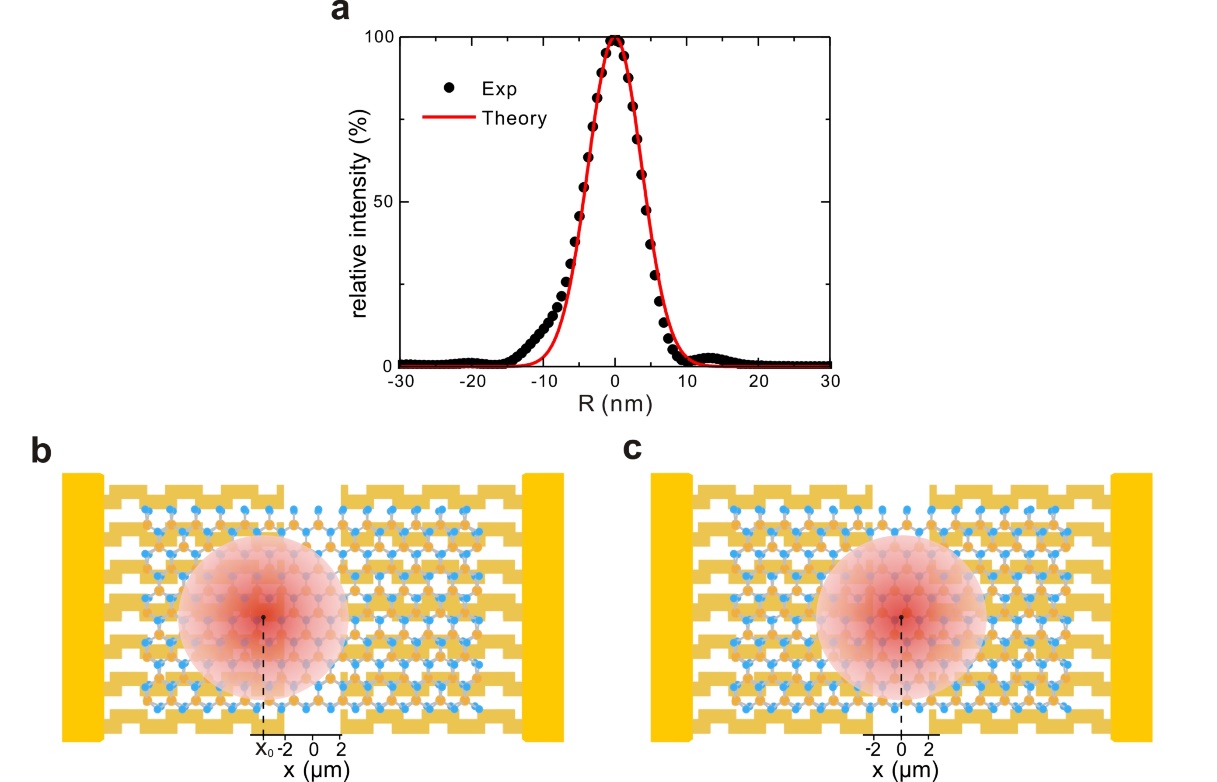


**Fig. S3. a**, Measured profile of the light spot and theoretical prediction based on the 2D Gaussian function. The FWHM of the measured profile is about 8.84 μm. **b-c,** Diagrams of the light spot positions for the ultrahigh-CPER mode and the balanced mode, respectively. The separation between the two photosensitive regions is 4 μm. The origin of the *x* axis is located at the channel center. *x*_0_ denotes the position of the light spot.

**3.2 Power distribution calculation**

Assuming the center of the light spot is located at *x*_0_, the power received by the left and right photosensitive region (*P_l_* and *P_r_*) are calculated to be:

$$P_{l}=aP_{d}=P_{0}c^{2}\int_{-\infty}^{(x_{0}-0.5L)/c} \exp\left(-\left(\frac{x}{c}\right)^{2}\right)d\left(\frac{x}{c}\right)\int_{-\infty}^{+\infty} \exp\left(-\left(\frac{y}{c}\right)^{2}\right)d\left(\frac{y}{c}\right)$$

$=\left(\frac{\pi}{2}\right)P_{0}c^{2}erfc\left(\frac{\left(0.5L+x_{0}\right)}{c}\right)$ (S11)

$$P_{r}=bP_{d}=P_{0}c^{2}\int_{x_{0}+0.5L/c}^{+\infty} \exp\left(-\left(\frac{x}{c}\right)^{2}\right)d\left(\frac{x}{c}\right)\int_{-\infty}^{+\infty} \exp\left(-\left(\frac{y}{c}\right)^{2}\right)d\left(\frac{y}{c}\right)$$

$=\left(\frac{\pi}{2}\right)P_{0}c^{2}erfc\left(\frac{\left(0.5L-x_{0}\right)}{c}\right)$ (S12)

where *L* = 4 μm represents the separation between the two photosensitive regions, *P_d_* denotes the total power received by the two photosensitive regions:

$P_{d}=\left(\pi/2\right)P_{0}c^{2}\left(erfc\left(\left(0.5L+x_{0}\right)/c\right)+erfc\left(\left(0.5L-x_{0}\right)/c\right)\right)$ (S13)

and *a* or *b* is the percentage of *P_l_* or *P_r_* in *P_d_*. *a* + *b* = 100%.

**In the ultrahigh-CPER mode**, when *a*/*b* = CPER_0_, the equation for $x_{0}$ writes:

$\frac{(\frac{\pi}{2})P_{0}c^{2}erfc\left(\frac{\left(0.5L+x_{0}\right)}{c}\right)}{(\frac{\pi}{2})P_{0}c^{2}erfc\left(\frac{\left(0.5L-x_{0}\right)}{c}\right)}=\text{CPER}_{0}$ (S14)

Then, $x_{0}$ is solved as a function of CPER_0_.

**In the balanced mode,** *a*/*b* = 1, and then $x_{0}$ is solved to be 0, indicating that the light spot is centered in the channel. $P_{d}=\left(\pi/2\right)P_{0}c^{2}erfc\left(0.5L/c\right)\approx0.297P_{light}$.

**3.3 Enhancing the power utilization ratio by shrinking the channel**

In either the ultrahigh-CPER mode or the balanced mode, the power incident at the channel (*P*_loss_ = *P_light_* − *P_d_*) is wasted. Concerning the channel length as 4 μm and assuming CPER_0_ = 4, *P*_loss_ is about 34.6% of *P_light_*. And it is 40.6% of *P_light_* in the balanced mode. Shrinking the channel length can reduce *P*_loss_, and thus enhance the signal-to-noise ratio (SNR). When the channel length is shortened to 2 μm, *P*_loss_ in the weighted mode is reduced to 16.5%, and that in the balanced mode is reduced to 21%. When the channel length is shortened to 1 μm, *P*_loss_ in the weighted mode becomes 7.9%, and that in the balanced mode is reduced to 10.6%. As we discussed in the main text, shrinking the channel length enhances the SNR and thus also enhances the CPER and the NEΔ*χ*.

**3.4 Wavelength dependent CPER_0_ and light spot position**

The CPER_0_ is wavelength dependent due to the resonant behavior of the chiral plasmonic structure. As shown in Fig. S4, with CPER_0_ varying with wavelength, *x*_0_ is adjusted according to Eq. S14 to ensure that the detector dimer is in the ultrahigh-CPER mode.


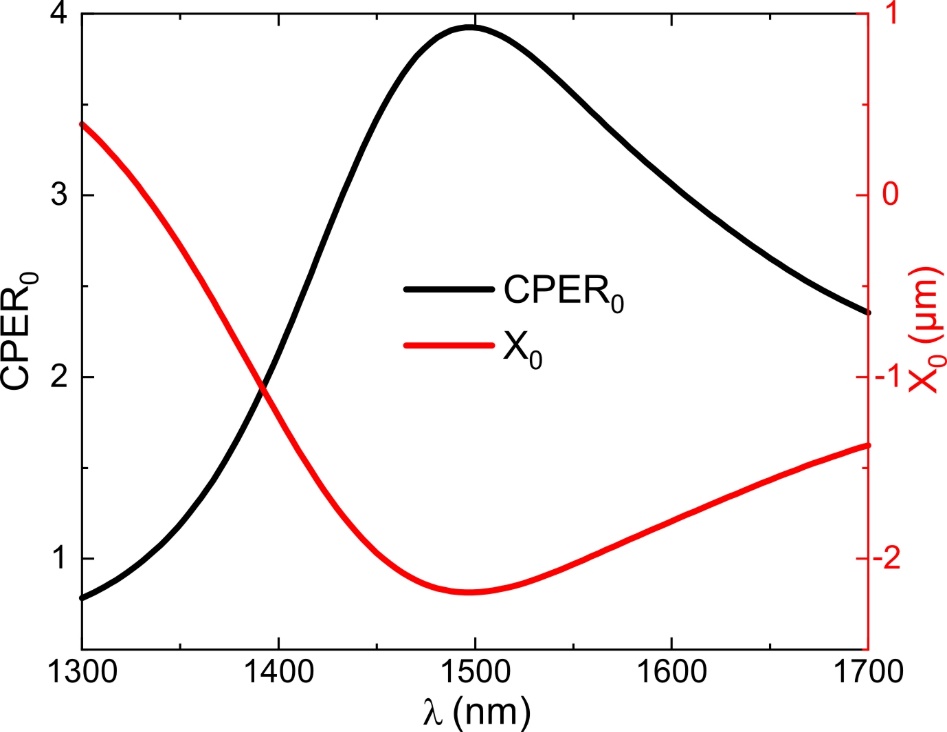


**Fig. S4.** Wavelength dependent $\text{CPER}_{0}$ of the Z-antenna array and the corresponding *x*_0_ for the detector dimer in the ultrahigh-CPER mode.

**3.5 How to achieve the required power distribution**

The required power distribution for the weighted mode or the balanced mode can be obtained by monitoring the photocurrent as the light spot in a specific polarization state moves across the device. This process is simulated as shown in Fig. S5a. The light spot takes a 2D Gaussian form. The photocurrent *i* equals *i*_1_ – *i*_2_, where *i*_1_ (*i*_2_) denotes the photocurrent generated from the left (right) photosensitive region and it is proportional to the simulated light absorptance of this region. The simulated photocurrent *i* at different polarization states, i.e. horizontal polarization (HP), left-handed circular polarization (LCP), right-handed circular polarization (RCP), and vertical polarization (VP), is presented in Fig. S5b. The ultrahigh-CPER mode corresponds to the light spot position of *i* = 0 at RCP or LCP. The balanced mode corresponds to the light spot position of *i* = 0 at HP or VP. Therefore, it is straightforward to achieve the required power distribution experimentally. And it is even possible to do it automatically just by a one dimensional motorized translation stage.


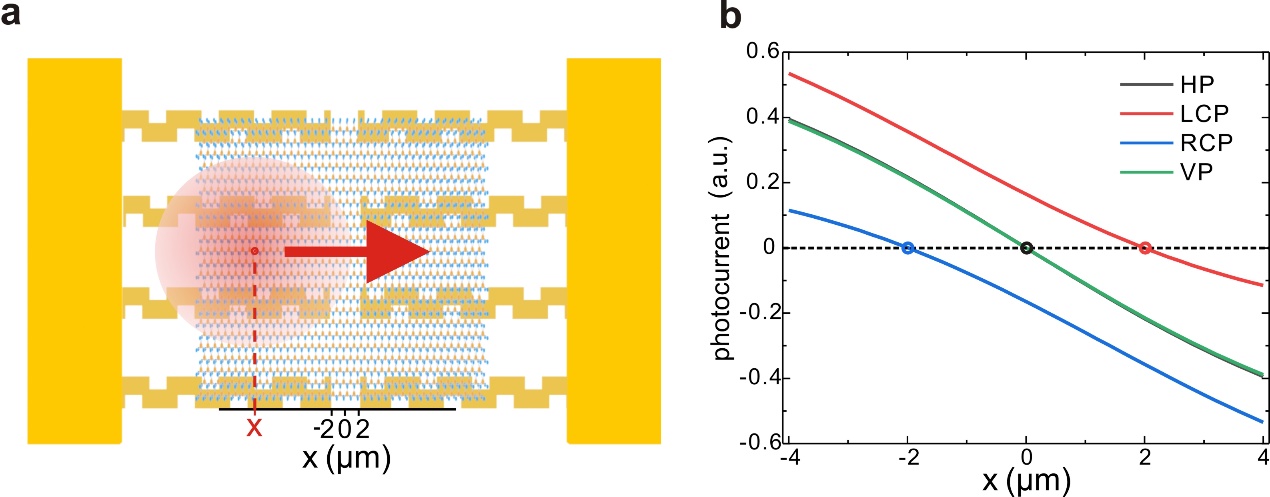


**Fig. S5**. **a,** Diagram of moving the light spot across the device along the *x* axis while monitoring the photocurrent for the required power distribution. **b,** Simulated photocurrent as a function of the light spot position. The black curve corresponds to the incident light at horizontal polarization (HP); the red curve corresponds to left circularly polarization (LCP); the blue curve corresponds to right circularly polarization (RCP); the green curve corresponds to vertical polarization (VP). The black curve overlaps the green curve since the chiral plasmonic structure responds similarly to HP and VP. The dots indicate the *i* = 0 position at each polarization state.

## Note 4. Derivation of the newly established CPER

Concerning the detector dimer in the ultrahigh-CPER mode, the photocurrent under LCP illumination (*χ* = *π*/4) writes:

$i\left(\pi/4\right)=aP_{d}R_{l}\left(\pi/4\right)-bP_{d}R_{r}\left(\pi/4\right)=aP_{d}R_{l}\left(\pi/4\right)\left(1-\frac{1}{\text{CPER}_{0}^{2}}\right)$ (S15)

since *a*/*b* = CPER_0_ and $R_{r}\left(\pi/4\right)=R_{l}\left(\pi/4\right)/\text{CPER}_{0}$. $aP_{d}R_{l}\left(\pi/4\right)=i_{1}$ denotes the photocurrent from the left photosensitive region of the detector dimer. Under RCP illumination, $i\left(3\pi/4\right)=0$, so the residual photoresponse is defined by the noise current *i*_noise_. Therefore, the CPER of the differential detectors can be written as:

$\text{CPER}=\frac{i_{1}\left(\pi/4\right)}{i_{noise}}\left(1-1/\text{CPER}_{0}^{2}\right)$ (S16)

*i*_noise_ is defined as $\sqrt{\int_{f_{1}}^{f_{2}} S_{i-noise}df}$, where *S_i_*_-noise_ is the noise spectral density, and *f* denotes the modulation frequency. *f*_1_ and *f*_2_ define the frequency band. When CPER_0_ = 1 corresponding to CPER = 0, the detector dimer is not able to discriminate circular polarizations. When CPER_0_ > 1 corresponding to CPER > 0, *R_l_* (*π*/4) > *R_l_* (3*π*/4), indicating that the left photosensitive region absorbs more LCP light than RCP light, and the detector dimer in the ultrahigh-CPER mode responds to LCP light and does not respond to RCP light. When CPER_0_ < 1 corresponding to CPER < 0, *R_l_* (*π*/4) < *R_l_* (3*π*/4), indicating that the left photosensitive region absorbs more RCP light than LCP light, and the detector dimer in the ultrahigh-CPER mode responds to RCP light and does not respond to LCP light. Moreover, the CPER is proportional to the SNR and positively correlated with CPER_0_. Therefore, unlike ordinary integrated circularly polarized detectors, even though the ellipticity discrimination ability of the chiral structure is poor, i.e. CPER_0_ is low, the CPER can still be high by improving the SNR.

## Note 5. Noise Analysis

At zero bias, the noise includes the 1/f noise, the light intensity noise, the generation-recombination (G-R) noise, the shot noise, and the thermal noise. The theoretical expressions of these types of noise are shown below. We fitted the theoretical expressions to the measured noise spectra, and reached a semi-analytical model that is consistent with the experimental results. Then, a discussion about the evolution of these noise components with the modulation frequency *f* is provided.

1. **thermal noise**

Thermal noise is caused by the thermal shock of electrons in conductors, and is present in all electronic devices. The noise spectral density of thermal noise writes:

$S_{\text{therm}}=\frac{4kT}{r}$ (S17)

where *k* = 1.38×10^-23^ J K^−1^ is the Boltzmann constant. *T* denotes the temperature, and it equals to 300 K in our study. *r* = 1.676×10^5^ Ω is the device resistance value.

1. **light intensity noise**

The light intensity noise originates from random fluctuations in light power^6^. The root mean square (RMS) of the light intensity writes:

$\text{RMS}\left(P\right)=\sqrt{\int_{0}^{T} \left(P-\bar{P}\right)^{2}dt/T}$ (S18)

where *P*(*t*) denotes the instantaneous intensity of the laser, and $\overline{P}$ denotes the averaged light intensity. According to our measurement of the light power:

$\frac{\text{RMS}\left(P\right)}{\bar{P}}\approx0.0729\%$ (S19)

Since:

$\text{RMS}\left(P_{d}\right)/\overline{P_{d}}=\text{RMS}\left(P\right)/\bar{P}$ (S20)

the RMS value of the photocurrent writes:

$\text{RMS}\left(i\right)=\text{RMS}\left(P_{d}\right)\cdot\left(aR_{l}-bR_{r}\right)=\left(\text{RMS}\left(P\right)/\bar{P}\right)\cdot P_{d}\cdot\left(aR_{l}-bR_{r}\right)$ (S21)

The noise spectral density of light intensity noise writes:

$\int_{f_{1}}^{f_{2}} S_{light}\left(f\right)df=\left(\frac{\text{RMS}\left(P\right)}{\bar{P}}\right)^{2}\cdot P_{d}^{2}\cdot\left(aR_{l}-bR_{r}\right)^{2}$ (S22)

where *f*_1_ = 0.03 Hz is the reciprocal of the measurement time, and *f*_2_ =166.5 Hz is half the sampling frequency of the laser power meter. Concerning a diode laser, the noise spectral density in low frequency range usually follows the 1/f behavior:

$S_{light}\left(f\right)=\frac{a_{0}}{f}\cdot P_{d}^{2}\cdot\left(aR_{l}-bR_{r}\right)^{2}=\frac{a_{0}}{f}\cdot i^{2}$ (S23)

$a_{0}$= 6.28×10^−8^ is a dimensionless coefficient determined by $\text{RMS}\left(P\right)/\bar{P}$. However, the light intensity noise does not necessarily follow Eq. S23. If the noise mainly comes from gas or fluid in the optical path, the expression of the noise is different.

1. **shot noise**

The shot noise mainly comes from the random fluctuation of photogenerated carriers^7^. The shot noise spectral density of the left photosensitive region and that of the right one write:

$S_{shot,l}=2q\cdot i_{l}=2q\cdot aP_{d}R_{l}$ (S24)

$S_{shot,\mathrm{r}}=2q\cdot i_{r}=2q\cdot bP_{d}R_{r}$ (S25)

where *q* is the unit charge. Since the photogenerated carrier fluctuations at the left photosensitive region and that at the right one are not correlated with each other, the overall shot noise is the sum of *S_shot,l_* and *S_shot,r_* :

$S_{shot}=S_{shot,l}+S_{shot,r}=2q\cdot(aP_{d}R_{l}+bP_{d}R_{r})$ (S26)

1. **1/f noise**

The 1/f noise caused by photocurrent fluctuations is proportional to the photocurrent. Following the famous expression by Hooge^8^, the spectral density of 1/f noise writes:

$S_{1/f}(f)=\frac{C_{1/f}i^{2}}{f^{\alpha}}$ (S27)

where *C_1/f_* is an empirical constant, $i=aP_{d}R_{l}-bP_{d}R_{r}$ is the photocurrent, and *α* is between 1 and 2. *C_1/f_* and *α* are determined by fitting the expression to the measured noise spectra.

1. **Generation-Recombination (G-R) noise**

The G-R noise is caused by the generation-recombination process of photogenerated carriers in the two photosensitive regions of the detector dimer under illumination^9^. The G-R noise spectral density of the left and right photosensitive regions are：

$S_{G-R,l}(f)=\frac{C_{G-R}\cdot i_{l}^{2}}{(1+(C_{f}\cdot f)^{2})}=\frac{C_{G-R}\left(aP_{d}R_{l}\right)^{2}}{(1+(C_{f}\cdot f)^{2})}$ (S28)

$S_{G-R,r}(f)=\frac{C_{G-R}\cdot i_{r}^{2}}{(1+(C_{f}\cdot f)^{2})}=\frac{C_{G-R}\left(bP_{d}R_{r}\right)^{2}}{(1+(C_{f}\cdot f)^{2})}$ (S29)

where *C_G-R_* and *C_f_* are two empirical constants. Since the G-R events are not correlated with each other, the overall G-R noise is the sum of $S_{G-R,l}(f)$ and $S_{G-R,r}(f)$:

$S_{G-R}(f)=S_{G-R,l}(f)+S_{G-R,r}(f)=\frac{C_{G-R}\left(\left(aP_{d}R_{l}\right)^{2}+\left(bP_{d}R_{r}\right)^{2}\right)}{(1+(C_{f}\cdot f)^{2})}$ (S30)

*C_G-R_* and *C_f_* are determined by fitting the expressions to the measured noise spectra.

1. **Total noise and fitting**

Through a comprehensive summary of these five types of noise, we can obtain the overall noise formula of the device:

$I_{noise}(f)=\sqrt{S_{therm}+S_{light}(f)+S_{shot}+S_{1/f}(f)+S_{G-R}(f)}$ (S31)

After fitting the above expression to the experimentally measured noise spectral density, the following parameters are determined: $C_{1/f}$ = $0.0875\cdot Hz^{2/3}$, *α* = $1.67$, $C_{G-R}$ = $1.05\times10^{-7}\cdot Hz^{-1}$, $C_{f}$ = $600\cdot Hz^{-1}$. Based on this analysis, the theoretical model agrees with the measured noise spectra in either the ultrahigh-CPER mode or the balanced mode at various ellipticity angles (Fig. S6).

As shown in Fig. S6, the 1/f noise dominates the 1-1000 Hz frequency range. The G-R noise dominates the 1000-10000 Hz frequency range. The other three types of noise are much smaller. At the silent state, where the photocurrent is zero, 1/f noise is greatly suppressed, and the device noise is decreased by 2 orders of magnitude in the low frequency range. As the frequency increases, the 1/f noise continuously decreases. And the noise decreasing rate increases as the *χ* angle deviates from the silent state. The G-R noise remains stable from 1-600 Hz, and decreases sharply after 600 Hz.


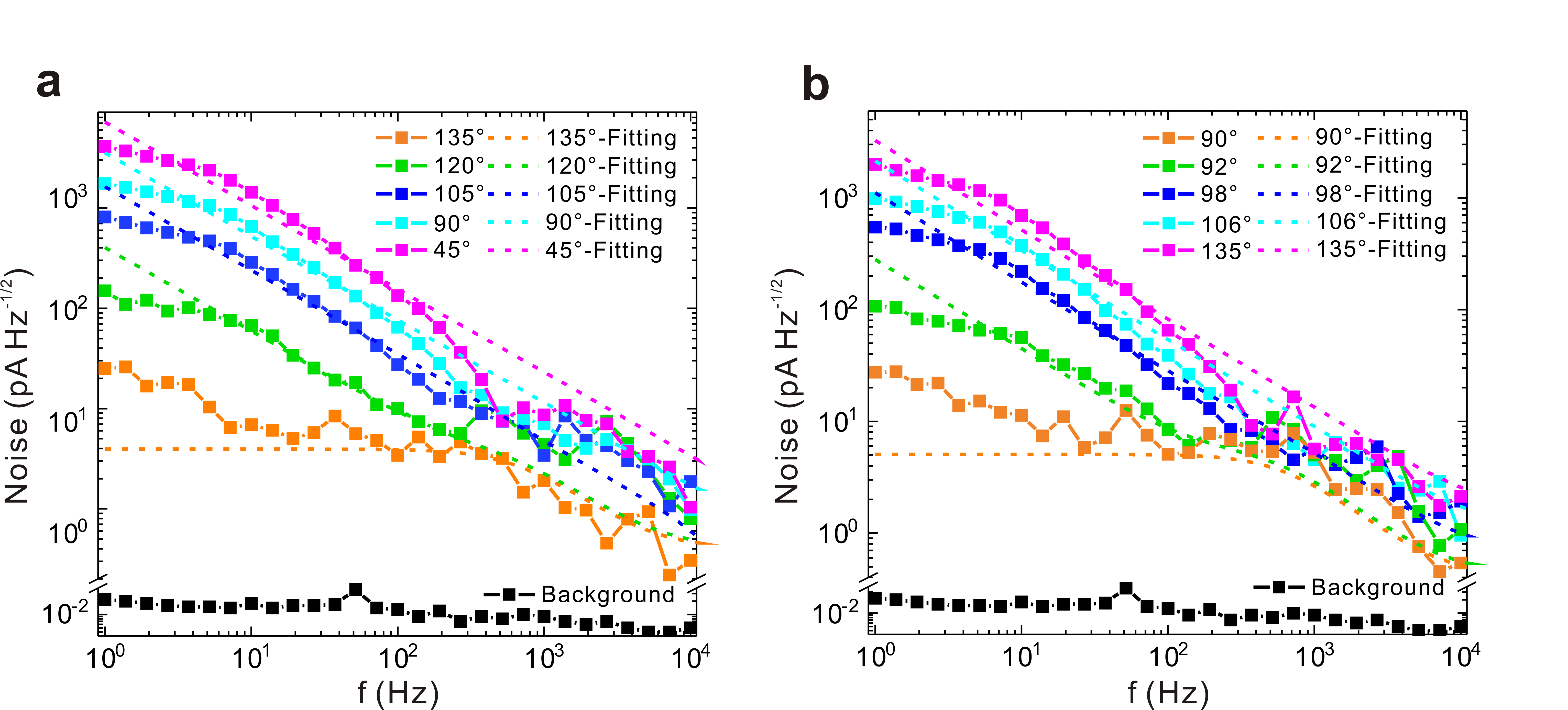


**Fig. S6. a,** Noise current spectra of the detector dimer at various *χ* angles in the ultrahigh-CPER mode, which is silent at *χ* = 135°. The light power *P*_light_ = 281 μw; the wavelength *λ* = 1550 nm; and the light spot position *x*_0_ = 2 μm. **b,** Noise current spectra of the detector dimer at various *χ* angles in the balanced mode. The silent state appears at *χ* = 90°. *P*_light_ = 281 μw; *λ* = 1550 nm; *x*_0_ = −0.025 μm. The squares represent the measured data and the dashed lines represent the fitted results based on the noise model.

Fig. S7 presents the noise spectral densities of all these five types of noise versus frequency under different conditions. The noise spectra densities in the ultrahigh-CPER mode are exhibited in Fig. S7a-c. Under RCP illumination (Fig. S7b), the device reaches the optoelectronic silent state corresponding to zero photocurrent and zero dark current. Since the 1/f noise is assumed to be mainly proportional to the current, it is eliminated. In addition, the laser intensity fluctuation caused photoresponses from the left and right photosensitive regions have the same magnitude and the opposite polarities in the silent state, so they cancel out. As a result, the total noise is significantly suppressed. Under LCP illumination, the device is in a responsive state. Then, all the five types of noise are present. The noise spectra densities in the balanced mode are exhibited in Fig. S7d-f. In this mode, the device reaches the silent state at light chirality transition points, such as the vertical polarization (VP), as shown in Fig. S7e. The silent state leads to elimination of 1/f noise and light intensity noise, and thus the suppression of the total noise. In contrast, under LCP illumination, the photocurrent is present and all the five types of noise show up.


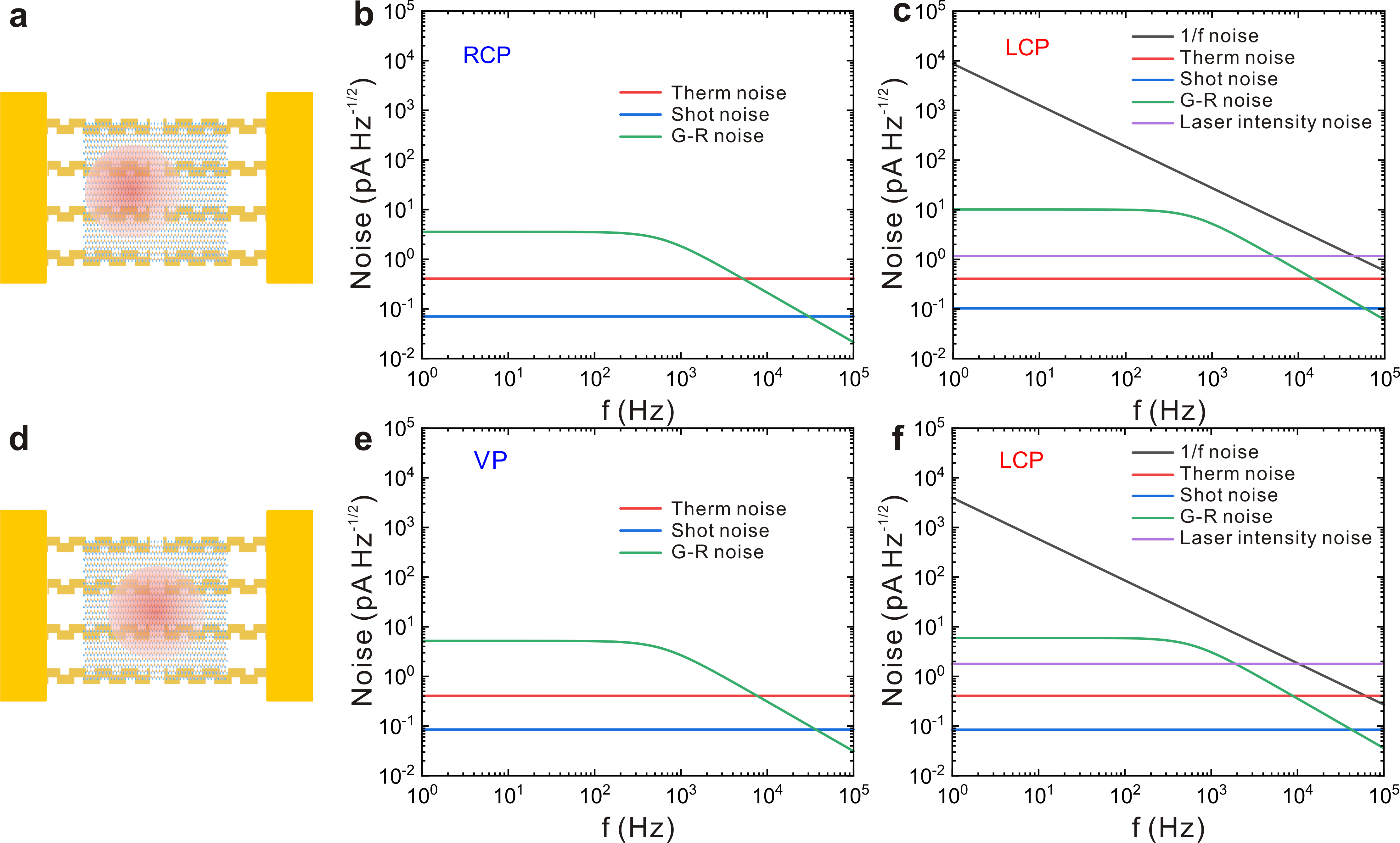


Fig. S7 **a,** Diagram of the device in the ultrahigh-CPER mode. **b-c,** Noise current spectra densities of the device in the ultrahigh-CPER mode under RCP and LCP illumination, respectively. **d,** Diagram of the device in the balanced mode. **e-f**, Noise current spectra densities of the device in the ultrahigh-CPER mode under VP and LCP illumination, respectively.

1. **Noise comparison between front-end photoresponse superposition and back-end data calculation**

The optoelectronic silent state, created by front-end photoresponse superposition, corresponds to complete absence of collective charge motion. Thus, the 1/f noise and the light intensity noise vanish in this state.

In comparison, if the two detector monomers (Fig. 1d in the main text) are not combined into a dimer, but separately connected to a computer doing the calculation of *i*_1_ − *i*_2_, there is no reduction but increase in noise. The total 1/f noise power is a sum of the 1/f noise powers of the two detector monomers. So is the light intensity noise. In this case, the 1/f noise spectral density writes:

$S_{1/f}(f)=\frac{C_{1/f}\left(\left(aP_{d}R_{l}\right)^{2}+\left(bP_{d}R_{r}\right)^{2}\right)}{f^{\alpha}}$ (S32)

Concerning a diode laser, the light intensity noise spectral density writes:

$S_{light}\left(f\right)=\frac{a_{0}}{f}\cdot\left(\left(aP_{d}R_{l}\right)^{2}+\left(bP_{d}R_{r}\right)^{2}\right)$ (S33)

in the absence of gas or fluid fluctuation. As shown in Fig. S8, the noise of the data obtained by back-end processing is much higher than that of the data obtained by front-end photoresponse superposition, regardless of whether the device in the weighted mode or the balanced mode.


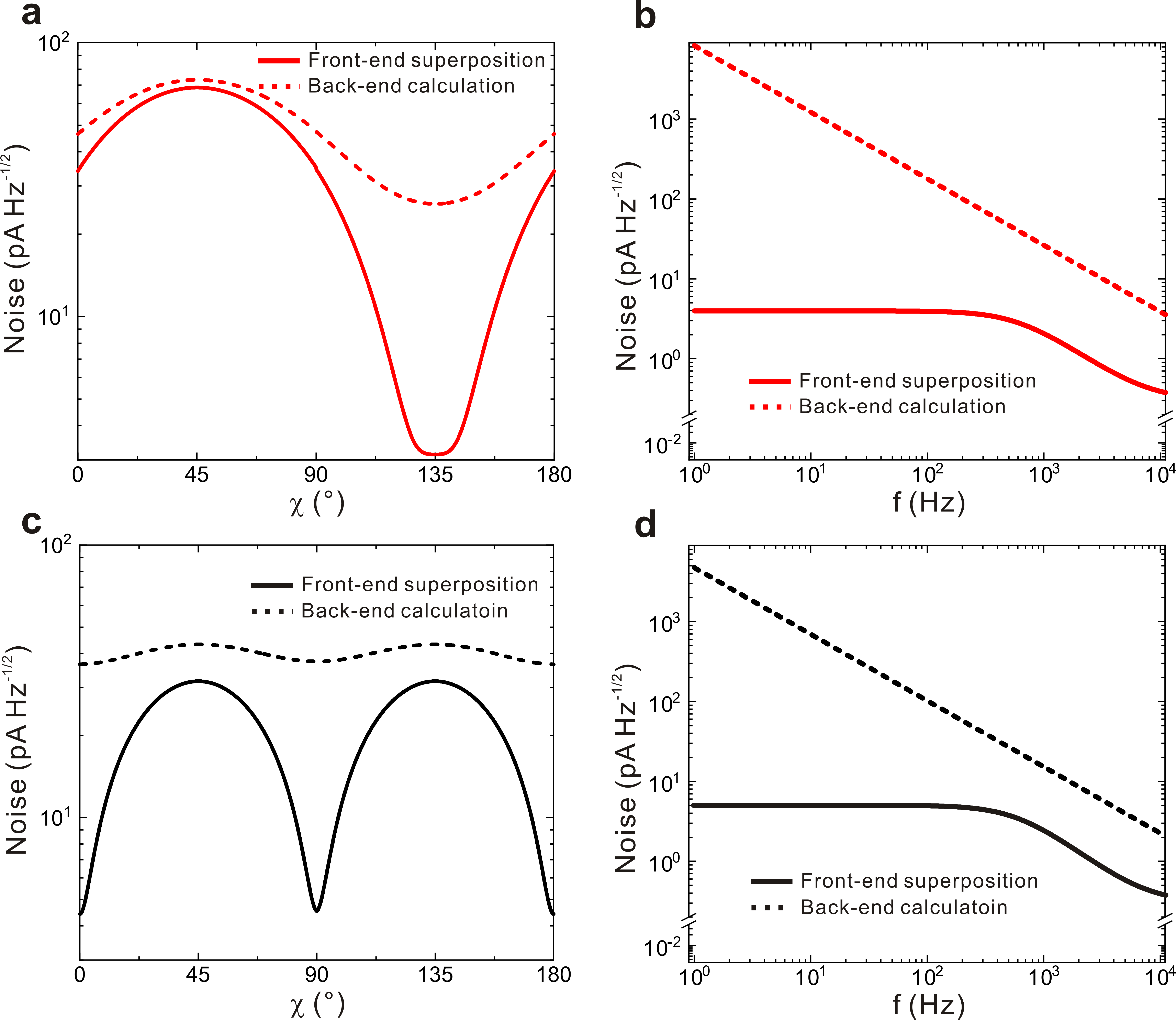


**Fig. S8. a,** Ellipticity angle dependent noise current of the detector dimer in the ultrahigh-CPER mode based on front-end superposition of the photoresponses from the two photosensitive regions (solid red line) and that based on back-end calculation of the two photoresponses (dashed red line). The silent state appears at *χ* = 135°. The modulation frequency is 333 Hz. **b,** Noise current spectra based on front-end photoresponse superposition in the silent state (*χ* = 135°) and that based on back-end data calculation at *χ* = 135°. **c,** Ellipticity angle dependent noise current of the detector dimer in the balanced state based on front-end superposition of the photocurrents from the two photosensitive regions (solid black line) and that based on back-end calculation of the two photocurrents (dashed black line). The silent state appears at *χ* = 90°. The modulation frequency is 333 Hz. **d,** Noise current spectra based on front-end photoresponse superposition in the silent state (*χ* = 90°) (solid black line) and that based on back-end data calculation at *χ* = 90° (dashed black line).

## Note 6. Optoelectronic silent state enhanced integrated circular polarization detectors with photovoltaic responses and PTE responses

**6.1 Integrated circular polarization detectors with photovoltaic responses**

If we replace MoS_2_ in our integrated circular polarization detector with a narrow-band semiconductor, the two photosensitive regions would generate photovoltaic responses. Fig. S9a shows a diagram of such a device. We assume that the band gap of the semiconductor is 0.34 eV, the conduction band edge is 4.72 eV from the vacuum level, and the doping level is 7×10^16^ cm^−3^. This assumption does not violate the universality, since many two-dimensional narrow-gap semiconductors, such as T-ZrSe_2_, T-HfSe_2_, PdSe_2_^10,11^, have the band structures similar to this assumption. The band distribution at the metal contact (Fig. S9b-c) is obtained by numerically solving the Poisson’s equation:

$\nabla\cdot\left(\varepsilon\nabla\psi\right)=-q(p-n-N_{A}^{-}+N_{D}^{+})$ (S34)

where *ε* denotes the permittivity of the semiconductor, *ψ* the potential, *q* the unit charge, *p* (*n*) the concentration of the holes (electrons), and $N_{A}^{-}$ ($N_{D}^{+}$) the concentration of the acceptors (donors). Here, the permittivity *ε* take a value of 9. The internal field of the Schottky junction will drive the photocarriers to form photovoltaic response. The photocarrier density is proportional to the light intensity, so the photovoltaic response is controlled by the Z-antenna in terms of circular polarization discrimination. The photoresponse of the device is simulated by solving the continuity equation:

$\nabla\cdot\mathbf{j}_{n}/\left(-q\right)=\left(G_{n}-R_{n}\right)$ (S35)

$\nabla\cdot\mathbf{j}_{p}/q=\left(G_{p}-R_{p}\right)$ (S36)

where the current consists of drift current and diffusion current:

$\mathbf{j}_{n}=qn\mu_{n}\mathbf{E}+qD_{n}\nabla n$ (S37)

$\mathbf{j}_{p}=qp\mu_{p}\mathbf{E}-qD_{p}\nabla p$ (S38)

**j***_n_* (**j***_p_*) denotes the current of electrons (holes), *G_n_* (*G_p_*) the generation rate of photogenerated electrons (holes), *R_n_* (*R_p_*) recombination rate of photogenerated electrons (holes), *μ_n_* (*μ_p_*) the mobility of electrons (holes), *D_n_* (*D_p_*) the diffusion coefficient of electrons (holes). The total current of the device equals **j***_n_* + **j***_p_*. In our simulation, the thickness of the semiconductor is assumed to be 5 nm. , where *n* = 3 and *k* = 0.01 is the real and imaginary part of the refractive index. *R_n_* and *R_p_* are derived through the Shockley-Read-Hall model, where the life time of electrons (holes) is assumed to be 100 ns. *μ_n_* and *μ_p_* are both 43.2 cm^2^ V^−1^ S^−1^. $D_{n,p}=\mu_{n,p}k_{B}T/q$. The light spot on the device takes a two-dimensional Gaussian form with the FWHM of 4 μm. The wavelength is 1.55 μm, and the light power is 80 μW. In the ultrahigh-CPER mode, the distributions of *G_n_* (*G_p_*) under LCP and RCP illumination are shown in Fig. S9d and e. Under LCP illumination, the photocarriers generated from the left photosensitive region are much more than those from the right photosensitive region, leading to a prominent photoresponse, as shown by the red line in Fig. S9h. Under RCP illumination, there are a number of photocarriers generated from the right photosensitive region since the right Z-antenna array is more likely to absorb RCP. And these photocarriers form a photocurrent canceling the photocurrent from the left photosensitive region, and then the residual photoresponse to RCP is eliminated. In the balanced mode, LCP light generates more photocarriers from the left photosensitive region than from the right photosensitive region (Fig. S9f). Horizontally polarized light generates equal number of photocarriers from the left and right photosensitive region (Fig. S9g), so the photocurrent becomes zero and the device is at the silent state. The *χ* dependent total photocurrent in the balanced mode is presented in Fig. S9h as the black line.


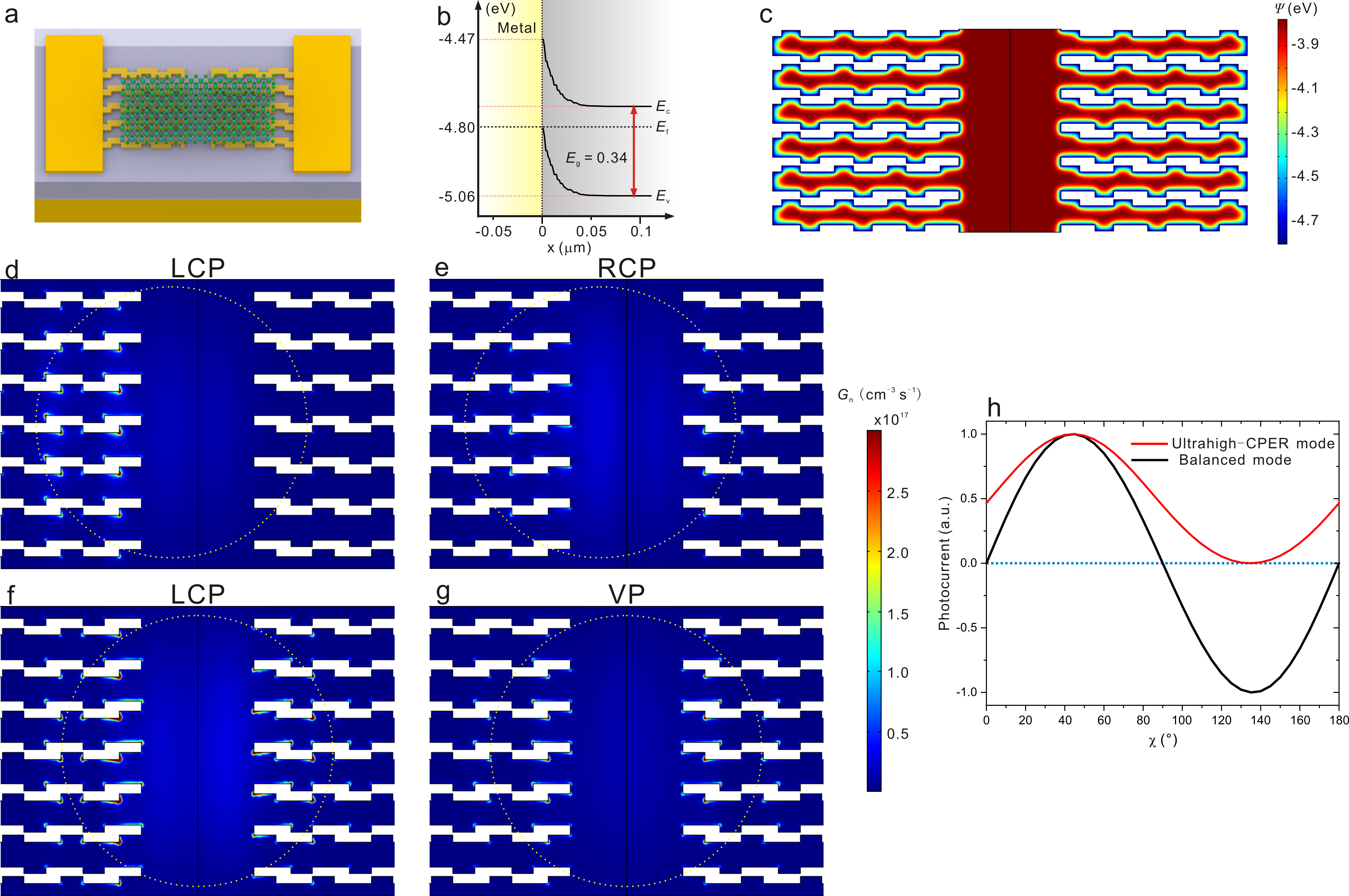


**Fig. S9. a**, Diagram of the silent state enhanced integrated circular polarization detector with photovoltaic response. **b**, Band diagram of the metal-semiconductor junction. **c**, The electrostatic potential distribution in the *x*-*y* plane without illumination. **d**-**e**, Distributions of photocarrier generation rate in the ultrahigh-CPER mode under LCP and RCP illumination. The dashed circle indicates the light spot position. **f**-**g**, Distributions of photocarrier generation rate in the balanced mode under LCP and VP illumination. The dashed circle indicates the light spot position. **h**, Normalized photocurrent versus light ellipticity angle *χ* in the ultrahigh-CPER mode and that in the balanced mode.

**6.2 Integrated circular polarization detectors with PTE responses**

A silent state enhanced integrated circular polarization detector with PTE response is shown in Fig. S10a. The active material is graphene. The fermi level of the graphene is 0.3 eV below the Dirac point. The geometry of the Z-antenna is designed to set the resonance at the wavelength of 3 μm. The band distribution near the metal contact is obtained by solving the Poisson’s equation. As shown in Fig. S10b-c, the graphene controlled by the metal is slightly *p*-doped and that in the channel is heavily *p*-doped. The variation of the doping condition induces a space-variant Seebeck coefficient:

$S\left(E_{f}\right)=-\frac{\pi^{2}k_{B}^{2}T}{3q}\frac{1}{\sigma}\frac{d\sigma}{dE_{f}}$ (S39)

*k*_B_ denotes the Boltzmann constant, *T* the temperature, and $\sigma=\sigma_{\min}\left(1+E_{f}^{2}/\Delta^{2}\right)$ the conductivity. The distribution of the Seebeck coefficient over the graphene is shown in Fig. S10d. The light absorption induces a temperature rise that causes current based on the PTE effect. When the incident light power is not too high, the temperature rise can be considered proportional to the field intensity ($\Delta T\left(x,y\right)\propto\left|\mathbf{E}\left(x,y\right)\right|^{2}$). The scale factor between $\Delta T\left(x,y\right)$ and $\left|\mathbf{E}\left(x,y\right)\right|^{2}$ can be estimated according to previous experiments about graphene PTE photoresponses^12^. The local photocurrent density writes $\mathbf{j}_{ph-loc}\left(x,y\right)=-\sigma S\left(x,y\right)\nabla T\left(x,y\right)$. According to the Shockley-Ramo effect^13^, the total photocurrent writes $I_{\text{PTE}}=A\iint\mathbf{j}_{ph-loc}\left(x,y\right)\cdot\nabla\psi\left(x,y\right)dxdy$, where $\psi$ denotes the weighting field and *A* is a prefactor which depends on device configuration. The weighting field can be calculated as the potential when one electrode is grounded and another electrode is set to be 1 V^13^. Since the local field is enhanced by the plasmonic resonance of the Z-antenna, the photocurrent is controlled by the Z-antenna in terms of circular polarization dependence. Fig. S10e-f present the temperature rise distributions in graphene for the device in the ultrahigh-CPER mode. The light spot on the device takes a two-dimensional Gaussian form with the FWHM of 11.3 μm. The wavelength is 3 μm, and the light power is 734 μW. The light spot inclines to the left for the ultrahigh-CPER mode. Under LCP illumination (Fig. S10e), the temperature rise mainly occurs in the left photosensitive region, since the left Z-antenna array is more likely to absorb LCP light and reflect RCP light while the right Z-antenna array behaves in the opposite way. Then, the PTE photocurrent mainly comes from the left photosensitive region. Under RCP illumination (Fig. S10f), the temperature rise in the left photosensitive region is balanced by that in the right photosensitive region, so the PTE photocurrent becomes zero and the device reaches the silent state. The *χ* dependent photocurrent of the device in the ultrahigh-CPER mode is shown by the red line in Fig. S10i. Fig. S10g-h present the temperature rise distributions in graphene for the device in the balanced mode. Under LCP illumination, the temperature rise in the left photosensitive region is more prominent than that in the right photosensitive region (Fig. S10g), so the photocurrent reaches a negative maximum. Under HP illumination, the temperature rise in the left photosensitive region is balanced by that in the right photosensitive region (Fig. S10h), so the photocurrent becomes zero and the device reaches the silent state. The *χ* dependent photocurrent of the device in the ultrahigh-CPER mode is shown by the black line in Fig. S10i.


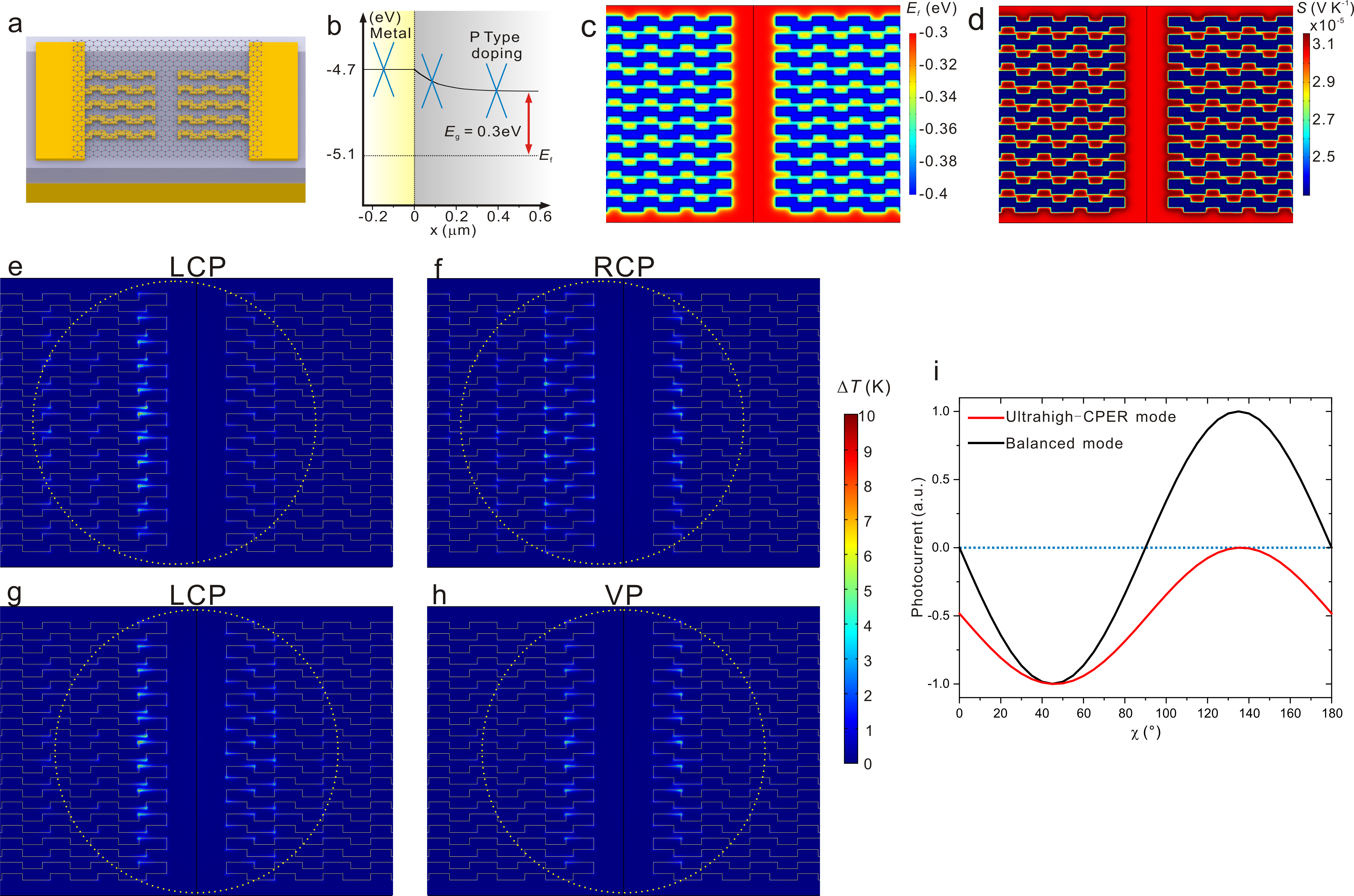


**Fig. S10**. **a**, Diagram of the silent state enhanced integrated circular polarization detector with PTE response. The geometry of the Z-antenna: *P_x_* = 1800 nm, *P_y_* = 1100 nm, *w* = 520 nm, *d* = 200 nm, *L* = 660 nm. The definitions of these parameters can be found in Fig. 2**b** in the main text. The thickness of the Z-antenna is 70 nm, and that of the Al_2_O_3_ spacer is 480 nm. **b**, Band diagram of the graphene under the metal and the graphene in the channel. **c**, Fermi energy distribution in the *x*-*y* plane without illumination. **d**. Distribution of Seebeak coefficient. **e**-**f**, Distributions of Δ*Τ* in the ultrahigh-CPER mode under LCP and RCP illumination. The dashed circle indicates the light spot position. **g**-**h**, Distributions of Δ*Τ* in the balanced mode under LCP and VP illumination. The dashed circle indicates the light spot position. **i**, Normalized photocurrent curve versus light ellipticity angle *χ* in the ultrahigh-CPER mode and that in the balanced mode.

## Note 7. Optoelectronic properties of the MoS_2_ device

Fig. S11a shows the I-V curves of our device under different gate voltages. The resistance of the device is about 167 kΩ at *V*_g_ = 0 V, and it decreases as *V*_g_ varies from negative to positive values. The gate voltage dependent current behavior is more clearly revealed by the transfer characteristic measurement (Fig. S11b). A small bias (*V*_ds_ = 10 mV) was applied to the source and the drain. The gate voltage dependent *I*_ds_ follows that of a typical n-channel FET. This result agrees with our expectation since the mechanically exfoliated MoS_2_ piece with a thickness of several nm is typically n-type^14, 15^. The channel is closed around *V*_g_ = −38 V. The on-off ratio is about 57 dB.

The optoelectronic characteristics of our device are shown in Fig. S11c-e. The incident light is LCP, and the wavelength is 1.55 μm. The light only illuminates the left photosensitive region of the detector dimer. Fig. S11c presents the modulated photocurrent at different light powers. The incident light is modulated at 1 Hz. The peak-to-peak power varies from 54 μW to 281 μW. In this power range, the photocurrent increases linearly with the light power. Our device operates under zero-bias, and thus there is no dark current. The none-zero base line in Fig. S11c is caused by the light that is not completely cut off during the modulation. The low-state power of the light is about 11 μW. Fig. S11d presents the bias dependent responsivity. It is natural that the responsivity increases with the bias. Fig. S11e shows that the photocurrent can be effectively regulated by the gate voltage. The tuning range is larger than 40 dB.

This behavior allows us to manipulate the responsivity by the gate voltage. As shown in Fig. S12, once the gate contact is split into the left and the right parts, the responsivities of the left and the right photosensitive regions can be tuned independently. This is a way other than light power distribution to tune the superposition coefficients *a* and *b*. The photocurrent of the detector dimer in the ultrahigh-CPER mode can also write:

$i=P_{l}a\left(V_{g1}\right)R_{l}-P_{r}b\left(V_{g2}\right)R_{r}$ (S40)

Here, *P_l_* = *P_r_*, corresponding to the flood illumination condition. $a\left(V_{g1}\right)R_{l}$ and $b\left(V_{g2}\right)R_{r}$ can be considered as the responsivities of the two photosensitive regions that can be tuned by gate voltages separately. *R_l_* and *R_r_* denote the responsivities at *V*_g_ = 0 V, and fulfill the relation $R_{l}\left(\chi\right)=R_{r}\left(\pi-\chi\right)$. In this case, by tuning *V*_g1_ and *V*_g2_ so that $a\left(V_{g1}\right)/b\left(V_{g2}\right)=\text{CPER}_{0}$, the detector dimer operates in the silent state for RCP light.


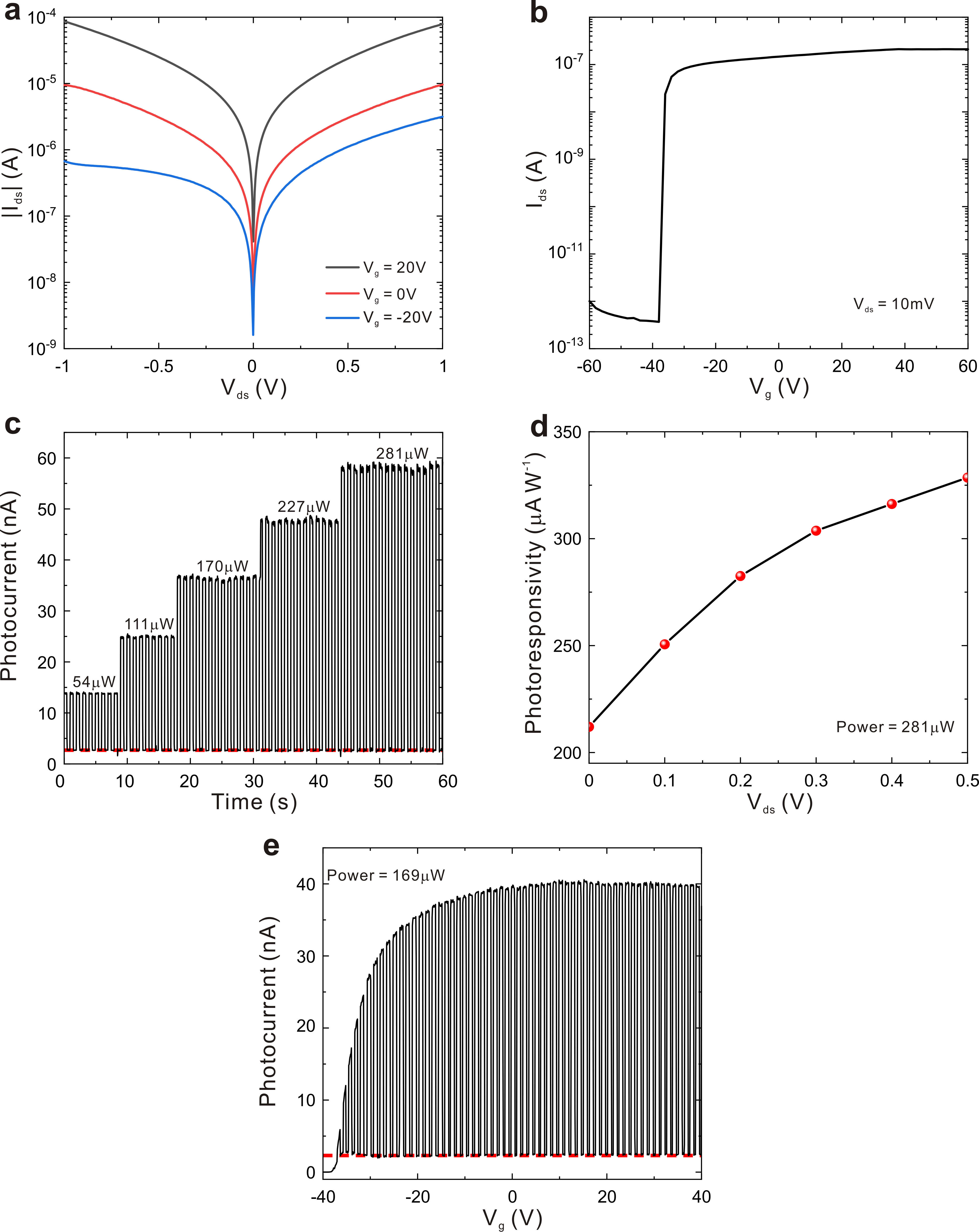


**Fig. S11. Optoelectronic properties of the device. a**, $I_{\text{ds}}\text{-}V_{\text{ds}}$ curves at different gate voltages. **b**, Transfer characteristics at *V*_ds_ = 10 mV. **c**, Modulated photocurrent at different laser power at 1Hz, *V*_ds_ = 0 V, *V*_g_ = 0 V. **d**, Bias voltage-dependent photoresponsivity. The incident light power is 281 μW. **e**, Modulated Photocurrent as a function of gate voltage at the incident light power of 169 μW. Except **a** and **b** tested under darkness, the wavelength of the light for all the measurements in the Fig.S10 is 1.55 μm.


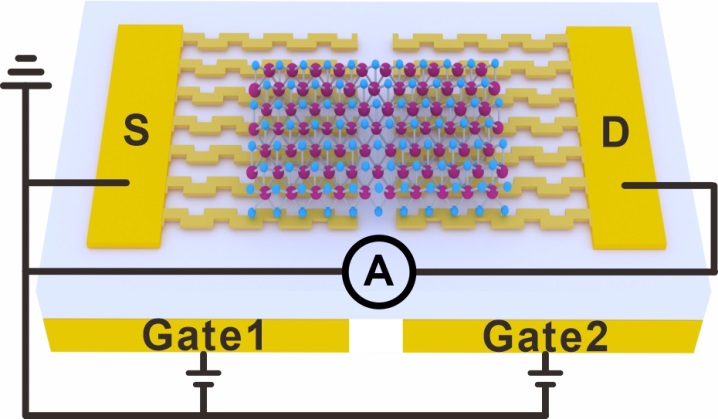


**Fig. S12. The detector dimer with a split Gate.** By tuning the electrostatic doping of the two photosensitive regions separately, the two responsivities (*aR_l_* and *bR_r_*) can be regulated.

Note 8. Immunity to disturbance at the silent state

Fig. S13 presents the photocurrent of the detector dimer in the balanced mode as a function of time and light ellipticity. Since the light ellipticity was controlled by rotating the HWP, the variation of *χ* correlates with time. The wavelength of the signal light was 1.55 μm, and the power was 8 μW. A modulated jamming light serving as the disturbance was superimposed on the signal. The modulation frequency of the jamming light was 3 Hz, and the power was 37 μW. At the *χ* angles other than the silent state (*χ* = 90°), the photocurrent was seriously interfered by disturbance. However, at the silent state, the disturbance is eliminated.

Therefore, at the silent state, the noise caused by the intensity fluctuation can be completely eliminated, even if the wavelength is within the ellipticity sensitive range of the Z-antenna. A potential application scenario is that during the detection of an elliptically polarized light, our device is immune to the disturbance from any horizontally or vertically polarized light.


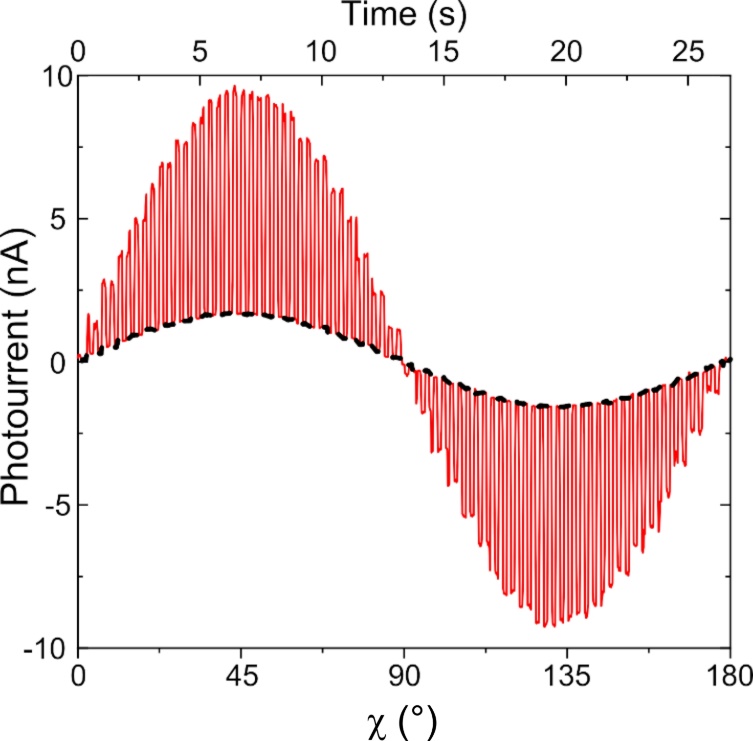


**Fig. S13.** Photocurrent recorded in real time. The time variation and the *χ* variation are correlated. A 3 Hz modulated jamming light with a power of 37 μW was superimposed on the single. The signal light has a power of 8 μW. The wavelength of either the signal light or the jamming light was 1.55 μm.

## Note 9. Comparison with commercial devices

The silent state enhanced integrated circular polarization detector is compared with commercial devices. At first, the silent state enhanced integrated circular polarization detector is compared with a combination of a quarter-wave plate (QWP), a linear polarizer (LP) and a detector about the CPER. The latter is somehow considered as the most reliable circular polarization detection method, since in most cases it defines the highest CPER a device can reach once the QWP, the LP, and the detector are of high quality. Concerning the CPER measurement in the QWP-LP method, circularly polarized light is at first transmitted through the QWP whose fast axis is 45° to the horizontal axis (*x*-axis), then through the LP that allows horizontally polarized light to pass, and finally to the detector. Ideally, the QWP turns LCP light into a horizontally polarized light that passes through the LP without any power loss, while it turns RCP light into a vertically polarized light that is completely blocked by the LP, leading to an infinite CPER. However, the QWP and the LP are not ideal, and the CPER becomes a finite value. Concerning a high-quality zero-order QWP like Thorlabs WPQSM05-1550 or SAQWP05M-1700, the retardance inaccuracy Δ*φ* is about *π*/500 to *π*/150. Concerning a high-quality LP like Thorlabs LPNIR050, the polarization extinction ratio (PER) is about 10^5^. Then, the Jones vector of an LCP light after passing through the QWP and the LP writes:

(S41)

The Jones vector of an RCP light after passing through the QWP and the LP writes:

(S42)

Where *M_QWP_* and *M_LP_* represent the QWP and the LP, respectively. *θ* = 45° represents the angle between the fast axis of the QWP and the *x*-axis. Then, the CPER is calculated to be:

$\text{CPER}=\frac{\left|a_{1}\right|^{2}+\left|a_{2}\right|^{2}}{\left|b_{1}\right|^{2}+\left|b_{2}\right|^{2}}\approx39\text{ to }47\text{ dB}$ (S43)

as listed in Table S1 first row. In comparison, the silent state enhanced detector dimer exhibits a CPER as high as 43 dB at the modulation frequency of 1 kHz with the incident light power as 281 μW (Table S1 5^th^ row). Therefore, the CPER obtained by our method is comparable to that obtained by the approach with the highest light ellipticity discrimination.

Although the QWP-LP method has the highest light ellipticity discrimination, the device based on that is not compact at all. Commercial circular polarizers can replace the combination of QWP and LP to shrink the size of the setup, but the larger retardance inaccuracy and smaller PER greatly reduce the CPER to around 30 dB (Table S1 2^nd^ and 3^rd^ rows).

When the QWP in the QWP-LP combination is rotating, a polarimeter is obtained. In addition to mechanical rotation of a QWP, the variation of the retardance can also be achieved by a liquid crystal variable retarder. A typical light ellipticity angle resolution of a commercial polarimeter is about 0.06°. In comparison, the light ellipticity angle resolution of our silent state enhanced integrated circular polarization detector is better than 0.009° when the modulated frequency is above 1000 Hz.

Concerning the device sizes, our silent state enhanced integrated circular polarization detector with a size about 19 × 42 μm is much more compact than a QWP-LP setup, a circular polarizer setup, or a polarimeter. The latter three all have benchtop sizes.

For linear polarization detection, there are commercial PER meters relying on rotating LPs. Table S1 6th-8th rows shows typical PER values and polarization angle resolutions. Although we do not find any CPER meters, it can be imagined that a CPER meter, as a counterpart of a PER meter, should rely on a rotating QWP, a fixed LP, and a detector. Therefore, the PER values and polarization angle resolutions of PER meters can be considered as a good reference for CPER values and ellipticity angle resolutions of CPER meters.

**Table S1**. The silent state enhanced integrated circular polarization detector versus commercial devices

| Model | CPER or PER | Ellipticity angle or polarization angle resolution | Size | Resolved polarization state |
| --- | --- | --- | --- | --- |
| Thorlabs  WPQSM05-1550 (SAQWP05M-1700) + LPNIR050 | 39 to 47 dB | / | Benchtop size | CPL |
| Thorlabs  **VC5-1550** | 33.5 dB | / | Benchtop size | CPL |
| Union Optic  VCP0010-1550^16^ | 29.5 dB | / | Benchtop size | CPL |
| Meadowlark  Polarimeter^17^ | < 47 dB | 0.06° | Benchtop size | LPL&CPL |
| This Work | **43 dB^*1^** | **< 0.009°^*1^** | **19 × 42 μm** | **CPL** |
| Techwin  PER-M500^18^ | 40 dB | 0.11° | Benchtop size | LPL |
| FIBERPRO ER2200^19^ | 45 dB | 0.1° | Benchtop size | LPL |
| LUNA ERM-202^20^ | 30 dB | 0.06° | Benchtop size | LPL |

*1: Power: 281 μW. Modulation Frequency: 1 kHz.

## Note 10. Experimental study on the graphene version of the silent-state-enhanced integrated circular polarization detector

We fabricated the silent-state-enhanced integrated circular polarization detector. Figure 14a shows the schematic diagram and the microscope photograph of the device. Except that the channel material has changed from MoS_2_ to graphene, other structures remain the same. The graphene device also works in the zero-bias mode. During the optoelectronic characterization, the wavelength of the incident light is 1.55 μm. According to many previous studies^12, 21-22^, the near-IR self-driven photoresponse at a graphene-contact junction is mostly attributed to the PTE effect. Figure 14b shows the *I*_ds_-*V*_ds_ curves for incident light at different power illuminating the Z-antenna array (right-handed) at the drain contact. The polarity of the photocurrent is opposite to that of the MoS_2_ device in the manuscript, since the photoresponse mechanism changes from hot electron injection to PTE. Figure 14c shows the waveform of the photocurrent at a modulation frequency of 1 Hz. As shown in the transfer characteristic (Fig. 14d), the graphene is slightly p-doped. The photoresponsivity can be regulated by *V*_g_. At *V*_g_ = −21.5 V, the responsivity reaches a maximum of 4.12 mA ∕ W, which is 19.4 times higher than that of the MoS_2_ device. The device operates in a linear range. The responsivity remains the same over the power range of 10^4^ (Fig. 14f). In the polarization-related tests, the CPER_0_ obtained by illuminating at left Z-antenna array (left handed) reaches 2.5 (Fig. 14g). Then, by tuning the position of the light spot, we demonstrate that the graphene device could also work in the ultrahigh-CPER mode and the balanced mode (Fig. 14h and 14i). Therefore, it is effective to improve the responsivity of our device by replacing MoS_2_ with graphene. Of course, the detection material is not restricted to graphene. Our scheme for circular polarization detection with ultra-high discrimination could also work with other detection materials with higher responsivities.


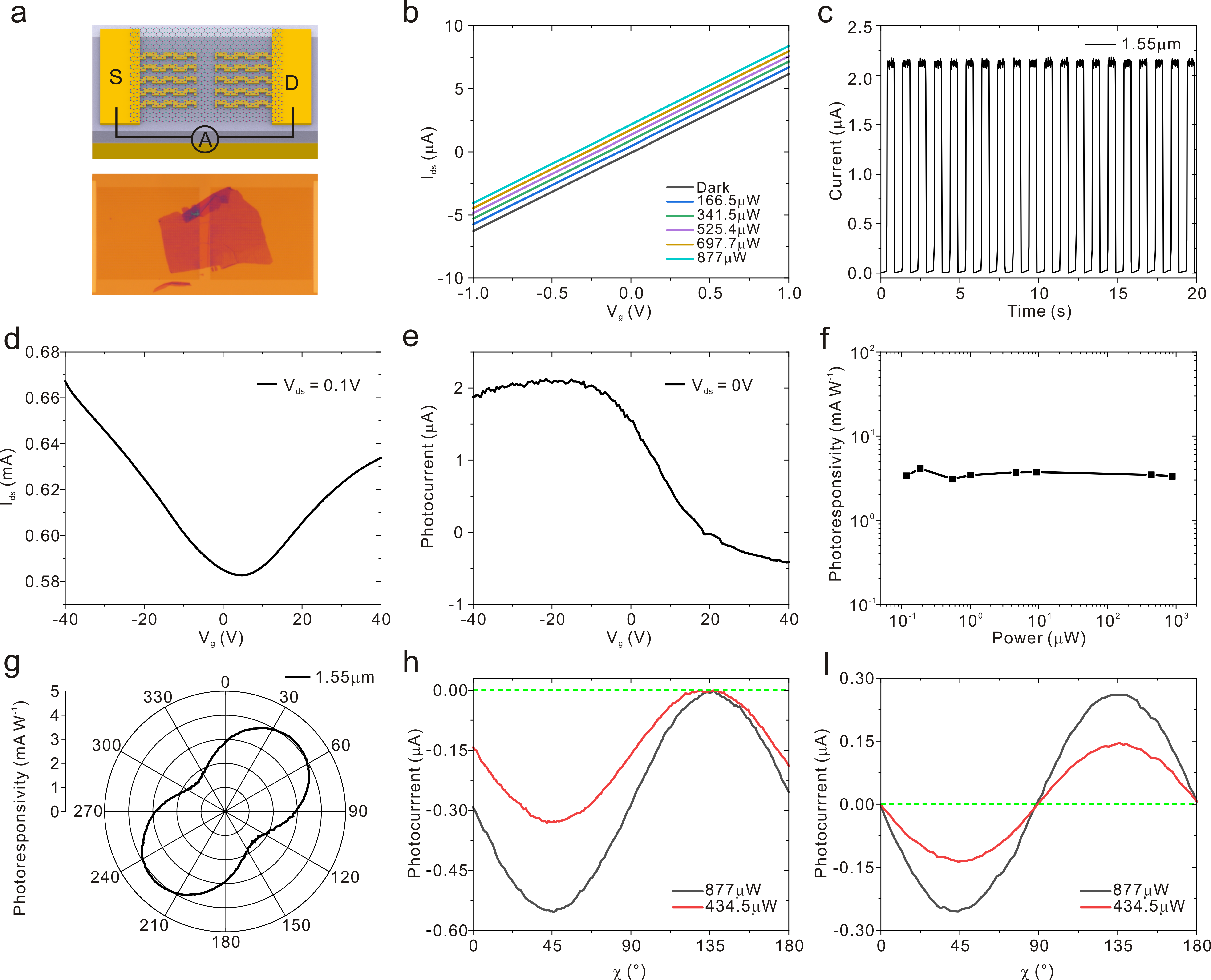


**Fig. S14**. **a,** Structure diagram and microscope photos of the device. Graphene was mechanically peeled off the HOPG and by transferred to the target location by dry transfer technology. **b,** *I*_ds_-*V*_ds_ curve for incident light at different power illuminating the Z-antenna array (right-handed) at the drain contact. *V*_g_ = 0 V. **c**, The photocurrent of the device at the modulation frequency of 1 Hz. **d**, Transfer characteristic of the graphene device at *V*_ds_ = 0.1 V. **e**, Responsivity regulated by *V*_g_ at the *V*_ds_ = 0 V. The light power is 643 μW. **f**, Responsivity as a function of light power. **g**, Ellipticity angle-dependent responsivity polar diagram. **h-i**, Ellipticity angle dependent photocurrents of the graphene device in the ultra-high CPER mode and in the balanced mode at the light power of 877 μW and 434.5 μW. For **b, c, e** and **f**, the incident light illuminates the right Z-antenna array (right-handed)**.** For **g-h**, the incident light illuminates the left Z-antenna array (left-handed)**.**

## Note 11. The thickness of MoS_2_

The thickness of MoS_2_ was measured by atomic force microscopy (AFM). As shown in Fig. S15, the measured thickness of MoS_2_ is 8.17 nm.


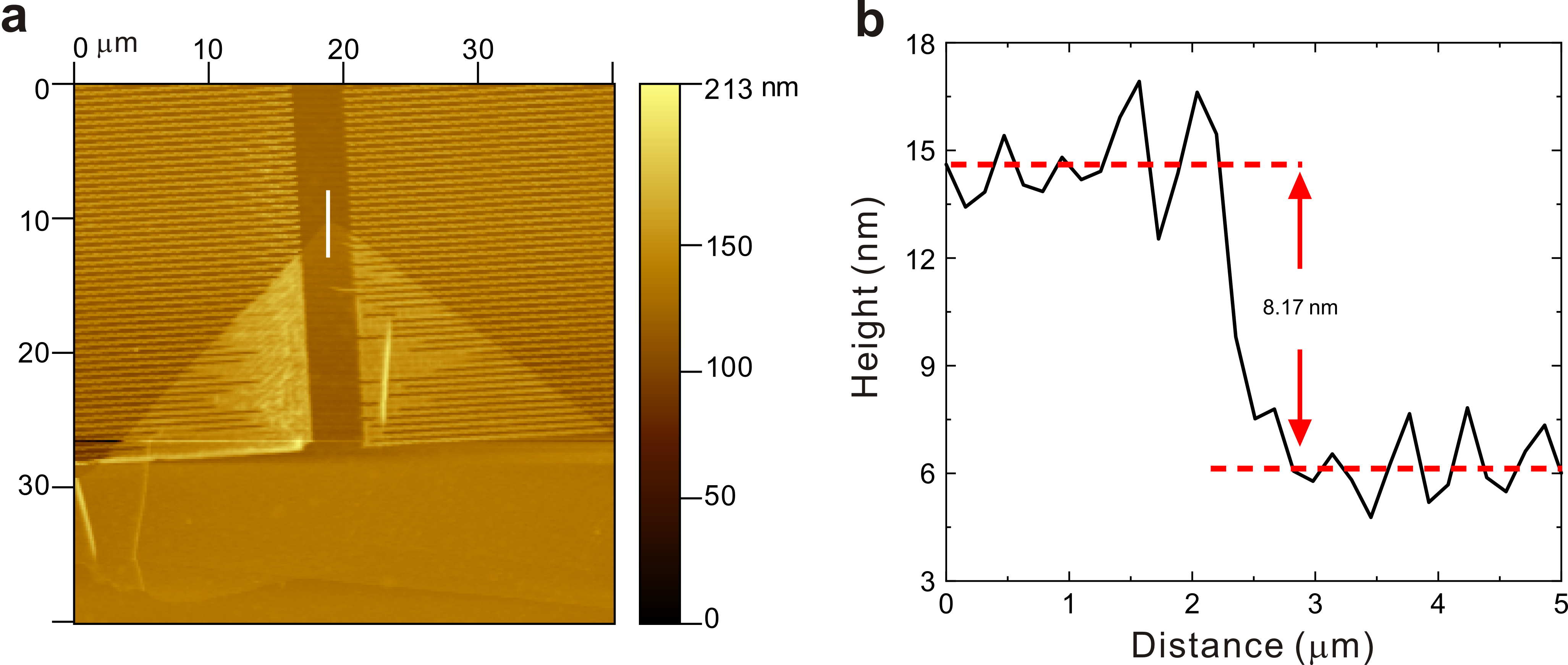


**Fig. S15. a**, AFM image. **b**, The relationship between distance and height from white line in **a**.

## References

1. Liu, Y. *et al*. Approaching the Schottky-Mott limit in van der Waals metal-semiconductor junctions. *Nature* **557**, 696-700 (2018).

2. Wang, J. L. *et al*. High Mobility MoS_2_ Transistor with Low Schottky Barrier Contact by Using Atomic Thick h-BN as a Tunneling Layer. Adv. Mat. **28**, 8302-8308 (2016).

3. Liu, H. W. *et al*. Epitaxial van der Waals contacts for low schottky barrier MoS_2_ field effect transistors. *Nano Res.* s12274-022-5229-y (2022).

4. Kaushik, N. *et al*. Schottky barrier heights for Au and Pd contacts to MoS_2_. *Appl. Phys. Lett.* **105**, 113505 (2014).

5. Moon, B. H. *et al*. Junction-Structure-Dependent Schottky Barrier Inhomogeneity and Device Ideality of Monolayer MoS_2_ Field-Effect Transistors. *ACS Appl. Mater. Inter.* **9**, 11240-11246 (2017).

6. Richardson, W. H., Machida, S., Yamamoto, Y. Squeezed photon-number noise and sub-poissonian electrical partition noise in a semiconductor laser. *Phys. Rev. Lett.* **66**, 2867-2870 (1991).

7. Gilbert, E. N., Pollak, H. O. Amplitude distribution of shot noise. *The Bell System Technical Journal* **39**, 333-350 (1960).

8. Hooge, F. N., Kleinpenning, T. G. M., Vandamme, L. K. J. Experimental studies on 1/f noise. *Rep. Prog. Phys.* **44**, 479-532 (1981).

9. Lauritzen, P. O. Noise due to generation and recombination of carriers in p-n junction transition region. *IEEE T. Electron Dev.* **ED-I5**, 770-776 (1968).

10. Zhang, C. X. *et al*. Systematic study of electronic structure and band alignment of monolayer transition metal dichalcogenides in Van der Waals heterostructures. *2D Mater.* **4**, 1-10 (2016).

11. Oyedele, A. D. *et al*. PdSe_2_: Pentagonal Two-Dimensional Layers with High Air Stability for Electronics. *J. Am. Chem. Soc.* **139**, 14090-14097 (2017).

12. Guo, S. K. *et al*. Enhanced infrared photoresponse induced by symmetry breaking in a hybrid structure of graphene and plasmonic nanocavities. *Carbon* **170**, 49-58 (2020).

13. Song, J. C. W. & Levitov, L. S. Shockley-Ramo theorem and long-range photocurrent response in gapless materials. *Phys. Rev. B* **90**, 1-6 (2014).

14. Radisavljevic, B., Radenovic, A., Brivio, J., Giacometti, V., Kis, A. Single-layer MoS_2_ transistors. *Nat. Nanotechnol.* **6**, 147-150 (2011).

15. Li, H.*, et al.* Fabrication of single- and multilayer MoS_2_ film-based field-effect transistors for sensing no at room temperature. *Small* **8**, 63-67 (2012).

16. https://www.u-optic.com/variable-circular-polarizer/en.html?siteid=2

17. https://secureservercdn.net/198.71.233.231/1v9.a14.myftpupload.com/wp-content/uploads/2022/03/

Polarimeter.pdf

18. https://www.sensing-laser.com/polarization-extinction-ratio-meter-per-m500-2-product/

19. https://ausoptic.com.au/mwdownloads/download/link/id/833/

20. https://lunainc.com/sites/default/files/assets/files/resource-library/ERM-202.pdf

21. Zhang, D. H. et al. Enhanced polarization sensitivity by plasmonic-cavity in graphene phototransistors. *J. Appl. Phys.* **126**, 074301 (2019).

22. Koppens, F. H. L. et al. Photodetectors based on graphene, other two-dimensional materials and hybrid systems. *Nat. Nanotechnol.* **9**, 780-793 (2014).
